# Supplementary material for: Cattle intestinal microbiota shifts following Escherichia coli O157:H7 vaccination and colonization
Source: PLoS One. 2019 Dec 5;14(12):e0226099. doi: 10.1371/journal.pone.0226099 (PMC6894827; doi:10.1371/journal.pone.0226099)
Supplement: S1 Table — Differences in bacterial community structure were examined by PERMANOVA analysis of the bacterial community structure (Beta-diversity). (DOCX) [file pone.0226099.s001.docx]

| **Comparison Groups** | **Sampling Weeks** | **Pseudo-f-statistics** | **p-value** |
| --- | --- | --- | --- |
| ^1^Vaccinated (Vx) and challenged (Ch) calves: Vx_E_-Ch vs Vx_C_-Ch | Wk 0 | 1.13 | 0.4 |
|  | Wk 1 | 1.23 | 0.17 |
|  | Wk 4 | 0.83 | 0.6 |
|  | Wk 5 | 0.82 | 0.74 |
|  | Wk 7 | 1.01 | 0.49 |
|  | Wk 8 | 1.16 | 0.14 |
|  | Wk 9 | 0.98 | 0.49 |
|  | Wk 10 | 1.11 | 0.34 |
|  | Wk 11 | 1.04 | 0.43 |
| All Vaccinated vs All Non-Vaccinated calves | Wk 0 | 1.0 | 0.39 |
|  | Wk 1 | 1.05 | 0.35 |
|  | Wk 4 | 1.1 | 0.28 |
|  | Wk 5 | 2.69 | 0.01 |
|  | Wk 7 | 1.63 | 0.05 |
| Non-Vaccinated and Non-Challenged (NonVx-NonCh) vs All Vaccinated and Challenged (Vx-Ch) calves | Wk 7 | 1.96 | 0.00 |
|  | Wk 8 | 1.67 | 0.01 |
|  | Wk 9 | 3.92 | 0.00 |
|  | Wk 10 | 3.7 | 0.00 |
|  | Wk 11 | 3.78 | 0.00 |
| Non-Vaccinated and Challenged (NonVx-Ch) vs All Vaccinated and Challenged (Vx-Ch) calves | Wk 7 | 2.33 | 0.00 |
|  | Wk 8 | 1.69 | 0.00 |
|  | Wk 9 | 3.2 | 0.00 |
|  | Wk 10 | 4.65 | 0.00 |
|  | Wk 11 | 3.07 | 0.00 |

**S1 Table. PERMANOVA Analysis of Microbial Diversity**

^1^ Vaccinated and challenged calves included two groups, one group vaccinated with the vaccine formulation containing adjuvant Emulsigen-D (Vx_E_-Ch) and the other group vaccinated with the vaccine formulation containing adjuvant Carbigen (Vx_C_-Ch).
