# Supplementary figures and images for: Cattle intestinal microbiota shifts following Escherichia coli O157:H7 vaccination and colonization
Source: PLoS One. 2019 Dec 5;14(12):e0226099. doi: 10.1371/journal.pone.0226099 (PMC6894827; doi:10.1371/journal.pone.0226099)

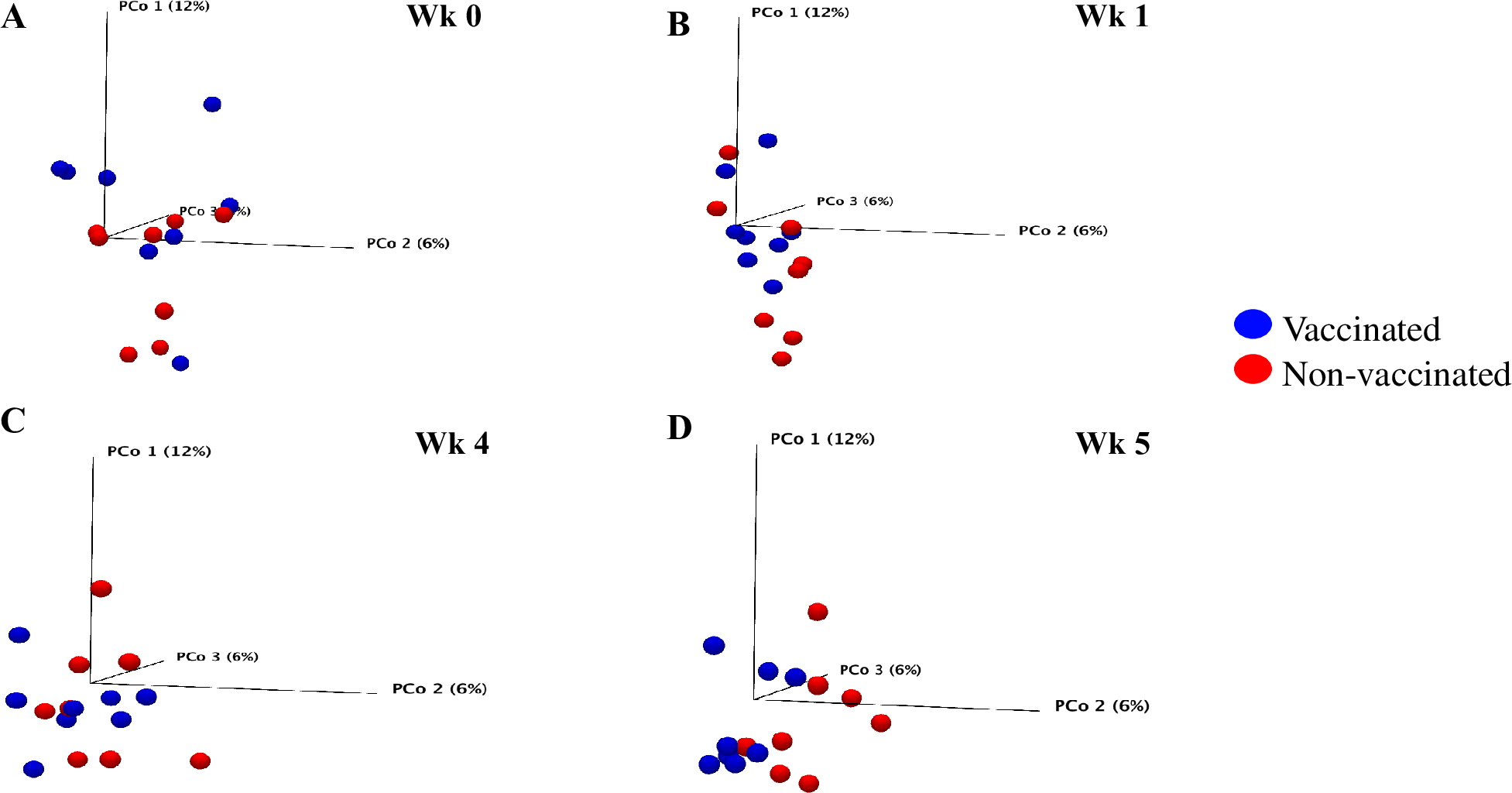

Supplement: S1 Fig — PCoA was performed for comparing bacterial community structure between vaccinated and non-vaccinated groups at sampling weeks 0, 1, 4 and 5, before the E. coli O157:H7 challenge was given. (TIF) [file pone.0226099.s002.tif]

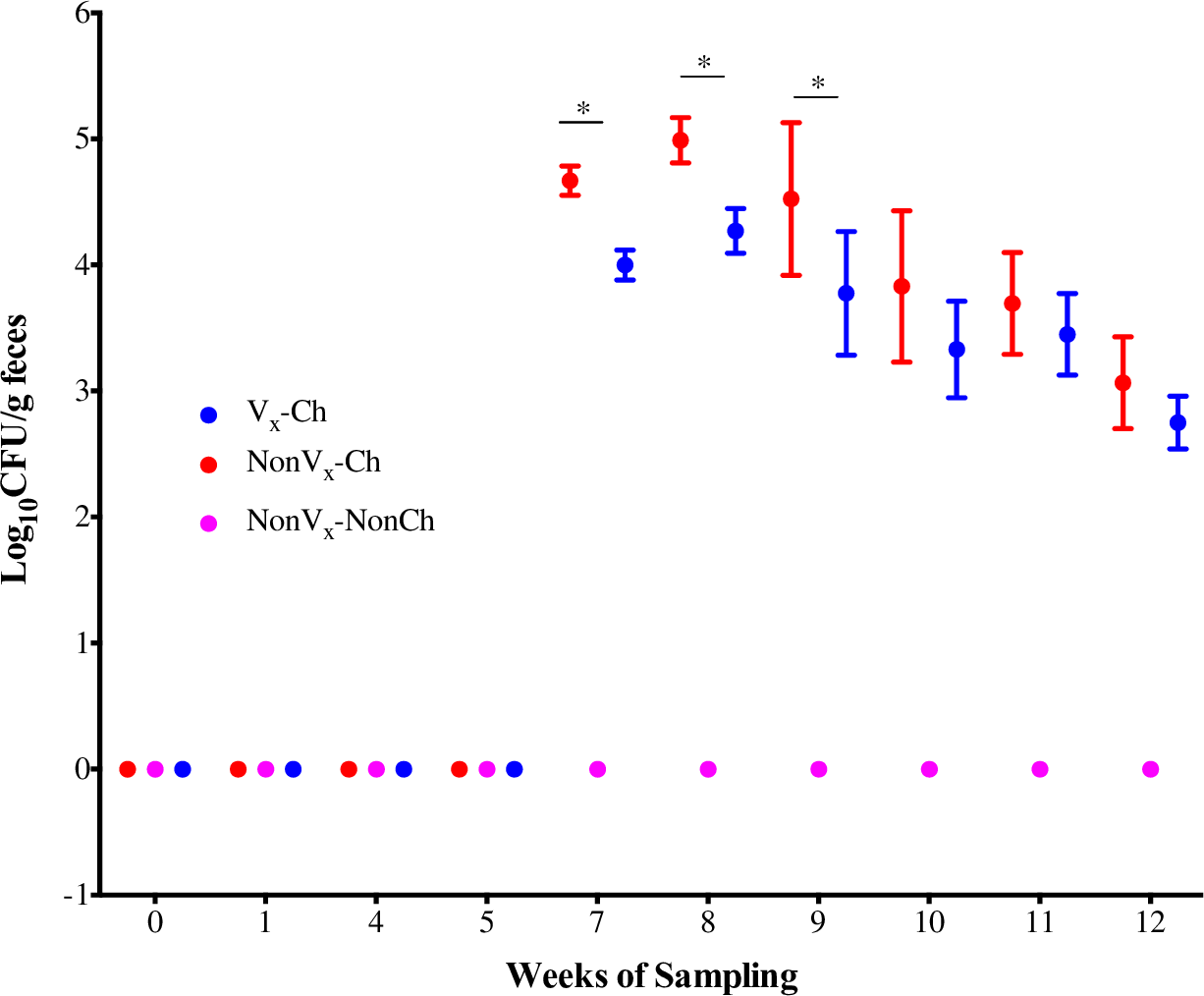

Supplement: S2 Fig — Number of bacteria shed was first Log transformed (represented as Log 10 CFU/g feces) and compared by ANOVA for difference between groups over a period of 12 weeks of sampling. (TIF) [file pone.0226099.s003.tif]

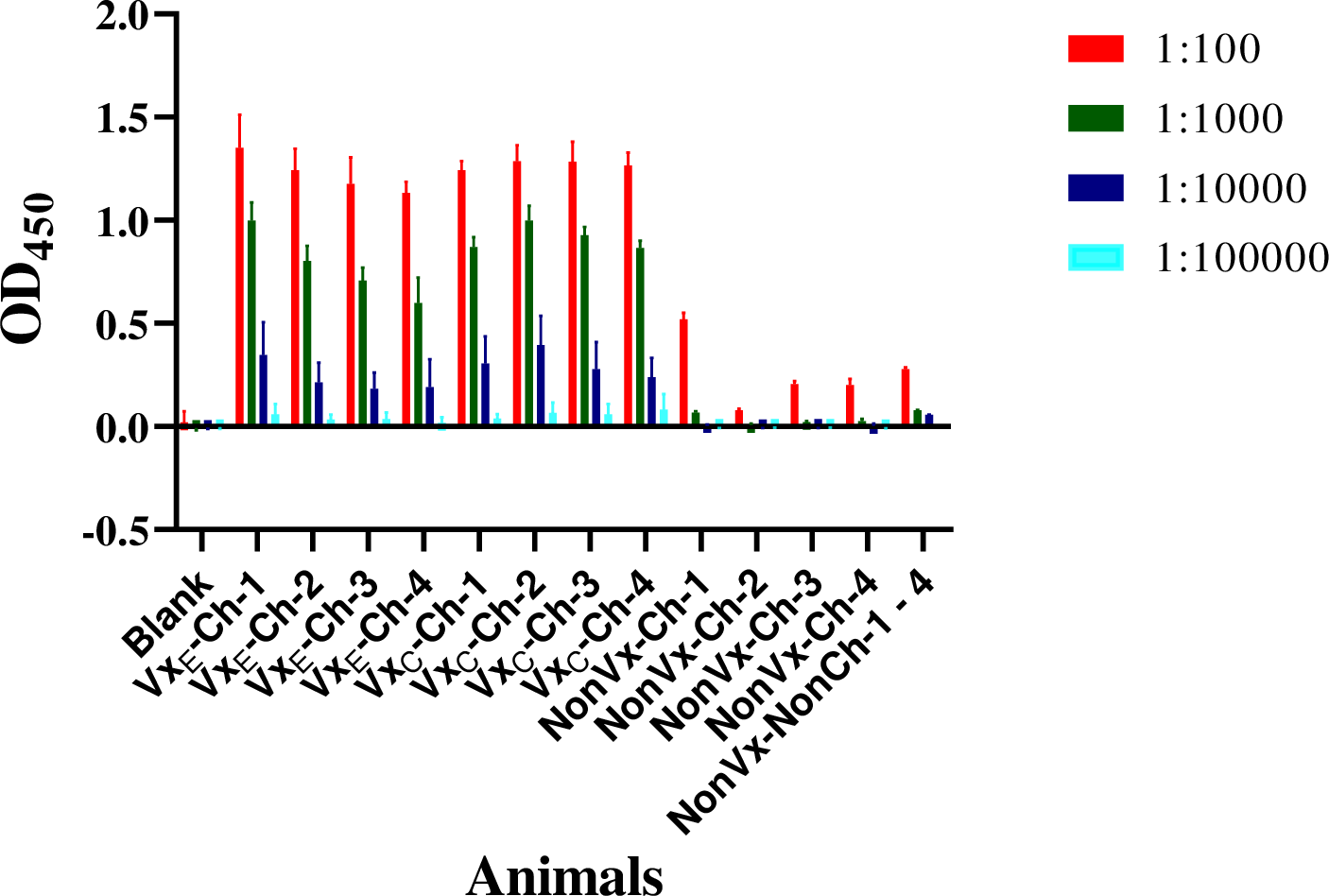

Supplement: S3 Fig — The serum IgG induced after vaccination was determined by reacting 10-fold serial dilutions of serum of each of the four calves from VxE-Ch, VxC-Ch, NonVx-Ch, and pooled sera of the four calves of NonVx-NonCh groups to the E. coli O157:H7 vaccine strain NADC 6597. Serum IgG levels are represented as Mean ± SD (shown as bars) of three replicate wells of the serum samples. Statistical analysis was performed using one way-ANOVA with multiple comparison of means. (TIF) [file pone.0226099.s004.tif]
