# Supplementary material for: Correction: Cattle intestinal microbiota shifts following Escherichia coli O157:H7 vaccination and colonization
Source: PLoS One. 2019 Dec 30;14(12):e0227403. doi: 10.1371/journal.pone.0227403 (PMC6936803; doi:10.1371/journal.pone.0227403)
Supplement: S2 File — (PDF) [file pone.0227403.s002.pdf]

PLOS ONE

RESEARCH ARTICLE

Cattle intestinal microbiota shifts following *Escherichia coli* O157:H7 vaccination and colonization

Raies A. Mir[1,2☯¤a], Robert G. Schaut[1,2☯¤b], Heather K. Allen[1], Torey Looft[1], Crystal L. Loving[1], Indira T. Kudva[1]*, Vijay K. Sharma[1]*

1 Food Safety and Enteric Pathogens Research Unit, National Animal Disease Center, Agricultural Research Service, U.S. Department of Agriculture, Ames, IA, United States of America, **2** Oak Ridge Institute for Science and Education (ORISE), ARS Research Participation Program, Oak Ridge, TN, United States of America

☯ These authors contributed equally to this work.
¤a Current address: Thermo Fisher Scientific, Alachua, Florida, United States of America
¤b Current address: Greenfield, Indiana, United States of America
* vijay.sharma@ars.usda.gov (VKS); indira.kudva@ars.usda.gov (ITK)

Check for updates

OPEN ACCESS

Citation: Mir RA, Schaut RG, Allen HK, Looft T, Loving CL, Kudva IT, et al. (2019) Cattle intestinal microbiota shifts following *Escherichia coli* O157:H7 vaccination and colonization. PLoS ONE 14(12): e0226099. https://doi.org/10.1371/journal.pone.0226099

Editor: Michael H. Kogut, USDA-Agricultural Research Service, UNITED STATES

Received: September 30, 2019

Accepted: November 18, 2019

Published: December 5, 2019

Copyright: This is an open access article, free of all copyright, and may be freely reproduced, distributed, transmitted, modified, built upon, or otherwise used by anyone for any lawful purpose. The work is made available under the Creative Commons CC0 public domain dedication.

Data Availability Statement: All relevant data are within the paper and its Supporting Information files.

Funding: The authors Raies Mir and Robert Schaut that are currently affiliated with Thermo Fisher Scientific, Alachua, Florida and Elanco International Inc., respectively, completed the work described in the manuscript PONE-D-19-27459 while employed as ORISE postdoctoral fellows at the National Animal Disease Center. Neither Thermo Fisher Scientific nor Elanco International had any role in

Abstract

Vaccination-induced *Escherichia coli* O157:H7-specific immune responses have been shown to reduce *E. coli* O157:H7 shedding in cattle. Although *E. coli* O157:H7 colonization is correlated with perturbations in intestinal microbial diversity, it is not yet known whether vaccination against *E. coli* O157:H7 could cause shifts in bovine intestinal microbiota. To understand the impact of *E. coli* O157:H7 vaccination and colonization on intestinal microbial diversity, cattle were vaccinated with two doses of different *E. coli* O157:H7 vaccine formulations. Six weeks post-vaccination, the two vaccinated groups (Vx-Ch) and one non-vaccinated group (NonVx-Ch) were orally challenged with *E. coli* O157:H7. Another group was neither vaccinated nor challenged (NonVx-NonCh). Fecal microbiota analysis over a 30-day period indicated a significant (FDR corrected, $p < 0.05$) association of bacterial community structure with vaccination until *E. coli* O157:H7 challenge. Shannon diversity index and species richness were significantly lower in vaccinated compared to non-vaccinated groups after *E. coli* O157:H7 challenge ($p < 0.05$). The *Firmicutes*:*Bacteroidetes* ratio ($p > 0.05$) was not associated with vaccination but the relative abundance of *Proteobacteria* was significantly lower ($p < 0.05$) in vaccinated calves after *E. coli* O157:H7 challenge. Similarly, Vx-Ch calves had higher relative abundance of *Paeniclostridium* spp. and *Christenellaceae* R7 group while *Campylobacter* spp., and *Sutterella* spp. were more abundant in NonVx-Ch group post-*E. coli* O157:H7 challenge. Only Vx-Ch calves had significantly higher ($p < 0.001$) *E. coli* O157:H7-specific serum IgG but no detectable *E. coli* O157:H7-specific IgA. However, *E. coli* O157:H7-specific IL-10-producing T cells were detected in vaccinated animals prior to challenge, but IFN-γ-producing T cells were not detected. Neither *E. coli* O157:H7-specific IgG nor IgA were detected in blood or feces, respectively, of NonVx-Ch and NonVx-NonCh groups prior to or post vaccinations. Both Vx-Ch and NonVx-Ch animals shed detectable levels of challenge strain during the course of the study. Despite the lack of

providing funding, such as salaries to these authors and/or research materials, when they completed this study at the National Animal Disease Center. Similarly, neither of these two companies had any role in the study design, data collection and analysis, decision to publish, or preparation of the manuscript.

Competing interests: We did not have any commercial affiliations with Thermo Fisher and Elanco International as indicated above in the Updated Funding Statement, but we do adhere to PLOS ONE policies on sharing data and materials.

protection with the vaccine formulations there were detectable shifts in the microbiota of vaccinated animals before and after challenge with *E. coli* O157:H7.

Introduction

Escherichia coli O157:H7 is a food borne pathogen acquired by ingestion of contaminated food, water or through direct contact with infected cattle or fomites [1]. Cattle are the primary reservoir for *E. coli* O157:H7, which preferentially colonizes at the rectoanal junction (RAJ) [2]. Although *E. coli* O157:H7 is not pathogenic to adult cattle [3], in humans it is associated with bloody diarrhea, hemorrhagic colitis, and hemolytic uremic syndrome (HUS, kidney failure) [4, 5]. A major risk factor for food contamination and human infections is fecal shedding of *E. coli* O157:H7 by cattle [6].

A mathematical model predicted an 80% reduction in the number of human illnesses if fecal shedding of *E. coli* O157:H7 by cattle could be reduced by 50% [7]. A meta-analysis of *E. coli* O157:H7 vaccination data also suggested that vaccination is an effective strategy for reducing fecal shedding of *E. coli* O157:H7 by cattle [8]. Frequency, duration, and intensity of *E. coli* O157:H7 shedding were significantly reduced in cattle vaccinated with either a siderophore receptor and porin protein (SRP)- or type III secreted proteins (T3SS)-based vaccine [9, 10]. Although variable efficacy is reported for a single-dose of a vaccine against *E. coli* O157:H7 (especially Shiga toxin 2 containing strains) [11], a three-dose SRP vaccine regimen was 85% effective in reducing *E. coli* O157:H7 shedding by an average of 1.7 $\log_{10}$ units in cattle feces [12]. Similarly, a three-dose heat-inactivated *hha* deletion mutant vaccine reduced the duration of fecal shedding of *E. coli* O157:H7 [13]. Previously, we demonstrated the efficacy of a two-dose vaccine formulation which stimulated high antibody titers and reduced the duration and magnitude of *E. coli* O157:H7 shedding in cattle feces [14]. This vaccine formulation contained a bacterin (chemically inactivated *hha* mutant strain of *E. coli* O157:H7) and a water-in-oil adjuvant [14]. Calves vaccinated with this vaccine formulation became culture negative for *E. coli* O157:H7 in three weeks after a challenge dose of 10^{10} colony forming units (CFU) of *E. coli* O157:H7 [14]. While protective, the above vaccine formulation exhibited vaccine site reactogenicity in calves, which warranted testing of additional vaccine formulations that would lack such negative effects at vaccination sites but would exhibit higher protective efficacy in terms of reducing colonization and fecal shedding of *E. coli* O157:H7 in cattle.

The native gut microbiota plays a significant role in the development and regulation of the immune response to pathogens, and an altered gastrointestinal (GIT) microbiota (dysbiosis) may affect the immunological outcome of vaccination [15, 16]. Analysis of the structure and functional profile of cattle GIT microbiota has gained pace recently [17], albeit with greater emphasis on the study of rumen microbiota and its modulation with different feeds and feed utilization [18–21]. Nevertheless, in humans and laboratory animals, specific members of the GIT microbiota influence antibody and cell-mediated immune responses [22, 23].

The three dominant phyla that comprise the GIT microbiota in cattle and almost all mammals are *Bacteroidetes*, *Firmicutes*, and *Proteobacteria* [24]. Lower abundance of *Firmicutes* relative to *Bacteroidetes* and *Proteobacteria* is an indicator of GIT microbiota dysbiosis [25, 26]. In cattle, the *Firmicutes*-to-*Bacteroidetes* (F:B) ratio was strongly correlated with daily milk-fat yield and parity of cows in milk, but the F:B ratio was not correlated with milk fat percentage or milk protein percentage [24, 27]. In the current study, we wanted to investigate the impact of vaccination for *E. coli* O157:H7 on cattle GIT microbiota, and subsequent shifts in

microbiota following *E. coli* O157:H7 challenge. Vaccine immunogenicity and efficacy of the formulations were also assessed, and results integrated with microbiota shifts. Collectively, vaccination was associated with shifts in the *Paeniclostridium* spp. and *Christenellaceae* R7 group of the GIT microbiota. Vaccination induced a peripheral anti-*E. coli* O157:H7 antibody response and *E. coli* O157:H7-specific T cell response dominated by IL-10 producers. Following challenge, peripheral T cells did produce *E. coli* O157:H7-specific IFN-γ, but it was not associated with protection.

Material and methods

Animal management and sample collection

Standard husbandry practices and veterinary care were applied to animals in the study. The research protocols used were approved by the USDA-ARS-NADC Institutional Animal Care and Use Committee. Jersey calves (6–8 months old) (n = 16) were tagged with a unique identification number and randomly assigned to one of the following treatment groups (n = 4 per group): 1) Non-Vaccinated and Challenged (NonVx-Ch); 2) Vaccinated (Emulsigen-D Adjuvanted vaccine) and Challenged (Vx_E-Ch); 3) Vaccinated (Carbigen adjuvanted vaccine) and Challenged (Vx_C-Ch); and 4) Non-Vaccinated and Not-Challenged (NonVx-NonCh). Adjuvants mentioned above were purchased from a commercial supplier (Phibro Animal Health Corporation, Teaneck, NJ). All the calves had free access to feed (pasture and hay) and water while housed outside in a pasture. A day before the challenge, calves were relocated from the pasture into a climate controlled BSL2 facility at the National Animal Disease Center (NADC) and housed in separate pens (4 animals per room per treatment group). Animals were fed twice daily with a maintenance diet of pelleted feed and alfalfa hay cubes and ad libitum access to water. Fecal samples (~10 grams) were collected from each animal by rectal palpation on day 0 (week 0) before vaccination, day 1 (week 1) at priming, day 21 (week 4) at boosting, day 42 (week7) at challenge, and once during weeks 8 to 12 (corresponding to days 49, 56, 63 and 70) post-challenge (Fig 1) resulting in a total of 144 samples from 16 animals (9 samples/animal). These samples were transported in sterile tubes, on wet ice, to the lab on the same day. An additional fecal sample was collected for culture from all animals at necropsy (Fig 1). Necropsy was performed after calves were humanely euthanized by the intravenous (jugular vein) injection of a barbiturate (sodium pentobarbital). Blood samples (from the jugular venipuncture) and additional fecal samples (n = 54) were collected from all calves at weeks 1, 4, 7, 8, 10 and 11 to identify and determine concentration of serum immune markers and fecal IgA, respectively **(Fig 1).** All calves were assigned to the Pain Category C since no clinical signs were expected following the vaccination, challenge, and sample collections. However, it was stated in the animal study protocol that the attending institution veterinarian will be consulted for appropriate handling of any calf exhibiting clinical or behavioral changes due to circumstances unrelated to the procedures (vaccination, challenge, and sample collections). All calves remained clinically healthy throughout the course of the study, except one calf developing an upper respiratory issue that was noticeable as a wheezing during the breathing. This calf was treated with an antibiotic (Draxxin) and an analgesic (Banamine) on the day the symptoms were first noticed while the analgesic was administered for the additional two days. On the third day following the treatment, calf's symptoms were marked resolved.

Vaccination and *E. coli* O157:H7 challenge

Streptomycin-resistant (Sm^R) *E. coli* O157:H7 strain NADC 6564 deleted of *stx2* and *hha* genes (NADC 6597) was used as a vaccine strain in the current study [14, 28]. The procedure for the construction of the vaccine strain NADC 6597 has been described previously [29]. To

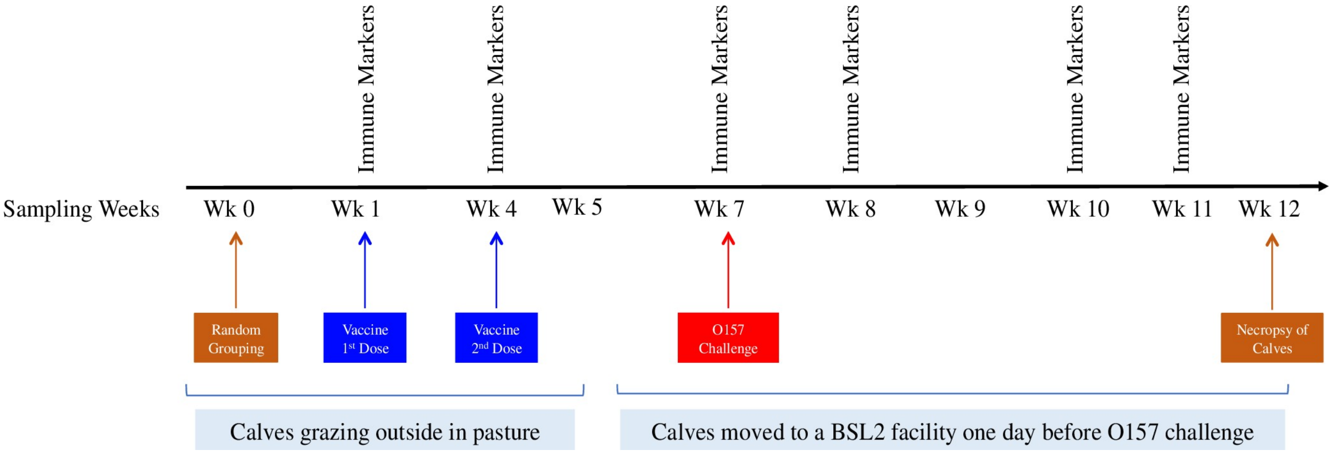

Fig 1. Experimental outline for animal vaccination and collection of fecal and blood samples. Calves (n = 16) were randomly assigned to one of four groups on week 0 (4 animals/group). Two groups of calves were vaccinated intramuscularly on week 1 followed by a booster dose on week 4. Vaccinated and challenged (Vx_E-Ch and Vx_C-Ch) and non-vaccinated but challenged (NonVx-Ch) calves were orally inoculated with *E. coli* O157:H7 on week 7 while the non-vaccinated and non-challenged (NonVx-NonCh) calves received sterile PBS. Fecal samples for culture and microbiota analysis were collected at weeks 0, 1, 4, 5, 7, 8, 9, 10 and 11. An additional fecal sample for culture was collected at necropsy. Blood samples for immune markers were collected at weeks 1, 4, 7, 8, 10 and 11.

https://doi.org/10.1371/journal.pone.0226099.g001

prepare the vaccine, an overnight culture of the vaccine strain NADC 6597 was diluted 1:100 in DMEM and grown (37˚C, 200 rpm) to an A_{600} of about 1.2. The bacterial culture was inactivated by adding formaldehyde (37% w/v) to a final concentration of 1.5% and incubation for 60 min at 37˚C with intermittent shaking as described previously [14]. Inactivated vaccine strain NADC 6597 (10^{10} cells in 1. 2 mL of phosphate-buffered saline (PBS)) was mixed with 0.8 mL of Emulsigen-D or Carbigen adjuvant and the resulting emulsions were used for vaccinating calves assigned to Vx_E-Ch and Vx_C-Ch groups, respectively. The vaccine formulation (2 mL) was injected in the muscle mass of the neck in front of the shoulder. Calves in the NonVx-Ch and NonVx-NonCh groups were mock vaccinated with 2 mL of PBS. Animals were boosted with the same preparations at day 21 (week 4) on the contralateral side of neck.

On day 42 (week 7), twelve calves, belonging to the Vx_E-Ch, Vx_C-Ch, and NonVx-Ch groups, were orally challenged with 10^{10} colony forming units (CFUs) of *E. coli* O157:H7 strain NADC 6564, and calves from the NonVx-NonCh group were mock-challenged with an equal volume of sterile PBS (**Fig 1**).

Detection and enumeration of *E. coli* O157:H7 strain NADC6564 (challenge strain)

Ten-grams of fecal samples (n = 144) after serial dilution in trypticase soy broth (TSB) (Difco Laboratories, Franklin Lakes, NJ) were plated on sorbitol-MacConkey agar containing streptomycin (100 μg mL^{-1}) and potassium tellurite (2.5 μg mL^{-1}) (SMAC-ST), directly or after incubation for 18 to 24 h, static at 37˚C (enrichment), to quantify fecal shedding of *E. coli* O157:H7. Fecal samples collected before the *E. coli* O157:H7 challenge were also serially-diluted in TSB with or without overnight enrichment and plated on sorbitol-MacConkey agar containing only potassium tellurite (2.5 μg mL^{-1}) to detect the naturally present *E. coli* O157:H7 in calves. Detection sensitivity for direct plating (without enrichment) was 10^3 colony-forming units (CFU) per 100 ml of fecal suspension (ten-gram feces in 90-mL of TSB). For enrichment plating, fecal samples were either assigned a value of one-log CFU or a value of 0 if these produced or did not produce *E. coli* O157:H7-sepcific colonies, respectively, on SMAC-ST plates.

Enzyme-Linked Immunosorbent Assay (ELISA) for detection of serum IgG and fecal IgA

E. coli O157:H7-specific serum IgG and fecal IgA were evaluated in blood and fecal extracts, respectively, collected from calves at various time points as shown on the study timeline (**Fig 1**). The ELISA procedures used for detection of IgG has been described previously [14] and it employed a commercially available kit (SeraCare, Milford, MA). Briefly, 96-well, medium-binding, round bottom plates were coated with an antigen consisting of 10^8 formalin inactivated cells of a non-pathogenic *E. coli* strain NADC 479, the vaccine *E. coli* O157:H7 strain NADC 6597, or the challenge *E. coli* O157:H7 strain NADC 6564 [14]. The wells of the antigen-coated plates were blocked with a blocking buffer and then filled with 100 μl aliquots of diluted pooled sera named Vx-Ch (pooled sera of 4 Vx_E-Ch and 4 Vx_C-Ch calves), NonVx-Ch (pooled sera of 4 NonVx-Ch calves), and NonVx-NonCh (pooled sera of 4 NonVx-NonCh calves). After 60 min incubation, the wells were washed with a kit-supplied washing buffer and then filled with 100 μl aliquots of a 10^{-4}-diluted HRP-conjugated anti-bovine IgG. The plates were washed after 60 min of incubation with HRP-conjugate and wells reacted with 100 μl of a two-component TMB. The reaction was stopped after 5 min of color development by adding 100 μl of stopping buffer and plates were read for absorbance at 405 nm wavelength (OD_{405}) in a SpectraMax Spectrophotometer (Molecular Devices, LLC, San Hose, CA). A commercially available Bovine IgA Quantitation kit (Bethyl Labs, Montgomery, TX) and the manufacturer's recommended procedure were used for detecting relative amounts of *E. coli* O157:H7-specific fecal IgA. Fecal IgA was detected in fecal extracts that were prepared as described previously [30]. Before using in ELISA, three groups of pooled fecal extracts were generated from feces collected on day 42 (week 7) immediately before *E. coli* O157:H7 challenge: Vx-Ch group (containing pooled fecal extracts of 4 Vx_E-Ch and 4 Vx_C-Ch calves), NonVx-Ch group (containing pooled fecal extract of 4 NonVx-Ch calves), and NonVx-NonCh group (containing pooled fecal extract of 4 NonVx-NonCh). Fecal extracts were analyzed against killed challenge strain NADC 6564 or a nonpathogenic commensal *E. coli* strain NADC 479 coated at 10^8 cells per well onto ELISA Immulon High Binding 2 plates (Thermo Scientific, Waltham, MA). After blocking the coated plate wells with a blocking buffer, 100 μl of a 10-fold diluted fecal extracts was added to blocked plates. Plates were washed five times with PBS containing 0.05% Tween-20 and anti-bovine IgA-HRP (1:10,000 dilution; Bethyl Labs, Montgomery, TX) was added to plate wells as per manufacturer's instructions. Ultra-TMB (Thermo Fisher Scientific) was added to plates after washing ten times with PBS+0.05% Tween-20. Plates were allowed to develop for 15 min and reaction stopped by adding 1M HCl. Plates were immediately read for absorbance at 450 nm (OD_{450}) on a SpectraMax Spectrophotometer.

Isolation of peripheral blood mononuclear cells and antigen recall response stimulation

Peripheral blood mononuclear cells (PBMCs) were isolated from 10 mL of blood as previously described [14]. Isolated PBMCs were counted for viable cells utilizing trypan-blue staining and plated at 10^6 cells/well into 96-well, tissue culture-treated, round bottom plates. Cells were then stimulated for 3 days with 5 μg of heat-inactivated, sonicated *E. coli* O157:H7 strain NADC 6564 lysate. Cells stimulated with 5 μg of pokeweed mitogen (Sigma Aldrich) were used as a positive control.

Flow cytometry intracellular staining

Cells were stained as previously described [30]. Briefly, at 16 hours prior to harvest, 10 μg/ml Brefeldin A (Sigma Aldrich) was added following manufacturer's recommendations for

intracellular staining. Cells were harvested and washed with FACS Buffer (PBS containing 1% Bovine Serum Albumin (Sigma Aldrich)), labeled for flow cytometry with primary-targeting antibodies at 21˚C for 15 min in PBS, washed with FACS buffer, and incubated with appropriate secondary antibodies for 15 min. Cells were washed with FACS buffer, fixed and permeabilized in saponin/formaldehyde solution (BD Fix/Perm, BD Biosciences) prior to adding antibodies to intracellular targets. Finally, cells were suspended in stabilizing fixative (BD Biosciences) and kept at 4˚C until data was acquired. Antibodies used were as follows: mouse anti-bovine γδTCR (TCR1-N24, δ-chain specific; clone GB21A, isotype IgG2b), CD4 (clone IL-A11A, isotype IgG2a), (Washington State University mAb Center, Pullman, WA); CD8-APC labeled (alpha-chain specific clone CC63), IFN-γ-PE labeled (clone CC302), (Bio-Rad Antibodies, Raleigh, NC); and anti-bovine IL-10-biotinylated labeled (clone CC320, Novus Biologicals, Littleton, CO). Secondary antibodies used were goat anti-mouse IgG1-AF488, IgG1-allophycocyanin, IgM-AF594, IgM-APC, IgG2b-PE-Cy7, IgG2b-AF350, and IgG2b-Cy5 (Southern Biotech, Birmingham, AL) Flow cytometry was performed on an LSR II (BD Biosciences) and analyzed using FlowJo v10 (FlowJo, LLC, Ashland, OR) software.

16S rRNA gene sequencing

DNA was extracted from fecal samples (n = 144) using the DNeasy PowerSoil kit (Qiagen, Germantown, MD). DNA yield and purity were evaluated on a Nanodrop (Life Technologies Corp., Grand Island, NY) and by electrophoresis on a 0.8% agarose gel. Previously described primers and conditions were used to amplify and sequence the V4 region of the 16S rRNA gene [31]. Each PCR mixture contained 17 μl *Accu*Prime *Pfx* SuperMix (Life Technologies Corp., Grand Island, NY), 5.0 μM each of the primers, and 25 ng of the template DNA. PCR settings included denaturation at 95˚C for 2 min and 22 cycles of (20 seconds at 95˚C, 15 seconds at 55˚C, 5 min 72˚C) amplification followed by final extension at 72˚C for 10 min. PCR amplicons were normalized using the SequalPrep™ Normalization Plate (96) Kit (Applied Biosystems Inc., Foster City, CA). Normalized amplicons were pooled and quantified using Kapa SYBR Fast qPCR (Kapa Biosystems, Wilmington, MA) and sequenced on a MiSeq Instrument using a MiSeq Reagent Kit v2 following manufacturer's instructions (Illumina, San Diego, CA). DNA from a mock community with defined composition [32] was also used to calculate sequencing error rates.

Data analysis

The sequences were analyzed using the Microbial Genomics Module (MGM) 1.6.1 (https://www.qiagenbioinformatics.com/solutions/microbial-genomics-solution/) (CLC Genomics Workbench, Qiagen Inc. Redwood City, CA) following the manufacturer's protocol for clustering of operational taxonomic units (OTUs). Specifically, the paired-end read data (forward and reverse sequences) were merged to create the highest quality sequences (trimmed to a fixed length of 250 bp) for clustering. The alignment settings were set as 1 for mismatch cost, 40 for minimum score, 4 for gap cost, and 5 as the maximum unaligned end mismatches. The OTUs were clustered at 97% similarity against the SILVA 16S rRNA small subunit reference database [33] and the metadata were added to the abundance table to aggregate samples based on metadata attributes.

The curated sequences were aligned in the MGM module using MUSCLE by the neighbor joining method and following the Jukes-Cantor model. This alignment was used to create a maximum likelihood phylogenetic tree. The phylogenetic tree and the OTU table describing the taxonomic differences among treatments and between weighted groups were used to calculate the Bray-Curtis dissimilarity and generate the PCoA plot. The difference in beta diversity

among the treatment groups was analyzed using the Permutation Multivariate Analysis of Variance (PERMANOVA) in the MGM module. This distance-based method tests the association of microbiome composition with any covariates of interest. The analysis and comparisons of alpha diversity (represented by measuring Shannon diversity index and Chao 1 species richness) between groups was carried out after the OTU tables were rarefied to the sample containing the lowest number of sequences (subsampled to 8,000 sequences per sample). Alpha-diversity measures were calculated in the MGM 1.6.1 and compared between vaccinated and non-vaccinated calves, using the Student's T-test. The difference in alpha diversity among the three groups (vaccinated: combined, Vx_E-Ch and Vx_C-Ch; mock: NonVx-NonCh; and challenged only: NonVx-Ch) of calves after the *E. coli* O157:H7 challenge was analyzed by one-way ANOVA and a cutoff value of 0.05 ($p < 0.05$) was selected to determine statistical significance (GraphPad Prism®, version 7.0c) of the output data. To determine the taxa that significantly differed between groups, we used differential abundance analysis (DAA) with the OTU table (at genera-level, taxon = Genus) as the input data. Prior to the analysis, OTU tables were rarefied to the sample containing the lowest number of sequences in each analysis. OTUs were assigned at the genus level, and genera with relative abundance of more than 0.1% of the total were used in DAA analysis. Post-hoc test (the false discovery rate, FDR) was used to determine significantly different (FDR p-value <0.05) taxa between groups.

Results

Vaccination did not induce clinical symptoms in calves

The primary and booster doses of the vaccine formulations were given intramuscularly in the neck region which was followed by an oral challenge with *E. coli* O157:H7 strain NADC 6564 or PBS. No overt clinical signs or negative health issues were reported after vaccination or oral challenge. All calves were sampled at designated time points and collection of these samples throughout the course of this study incurred no injury to the animals due to restraining or sample collection procedures.

Vaccination to *E. coli* O157:H7 was associated with shifts in microbial communities

Sequencing of the V4 region of the 16S rRNA gene in all 144 fecal samples resulted in 2.63 million reads, which after filtering and removing chimeras yielded 3,974 predicted Operational Taxonomic Units (OTUs). The OTU table and the sample information (metadata) was used to determine statistical differences in bacterial community structure (**S1 Table**). There was no significant difference in bacterial community structure between samples from the two vaccinated (Vx_E-Ch and Vx_C-Ch) groups throughout the study, so both were combined into a single group, called as a vaccinated-challenged group (Vx-Ch). No significant differences (p-value>0.05) were observed in bacterial community structure between the vaccinated and non-vaccinated groups at week 0, week 1 (samples collected before the vaccination) and at week 4 sampling (sampled before the booster-dose vaccine was given) (**S1 Fig**, **S1 Table**). But at week 5, one-week after the booster-dose was administered, the bacterial community structure was significantly different (p-value = 0.01) (**S1 Table**) between vaccinated and non-vaccinated groups (**S1 Fig**). Also, the bacterial community structure was different (p-value = 0.05) (**S1 Table**) between vaccinated and non-vaccinated groups on week 7 of sampling before the *E. coli* O157:H7 challenge (**Fig 2**). After oral challenge of calves with *E. coli* O157:H7 or PBS, the bacterial community structure was different between Vx-Ch and NonVx-Ch, and between Vx-Ch and NonVx-NonCh groups (p-value < 0.05) (**Fig 2**, **S1 Table**).

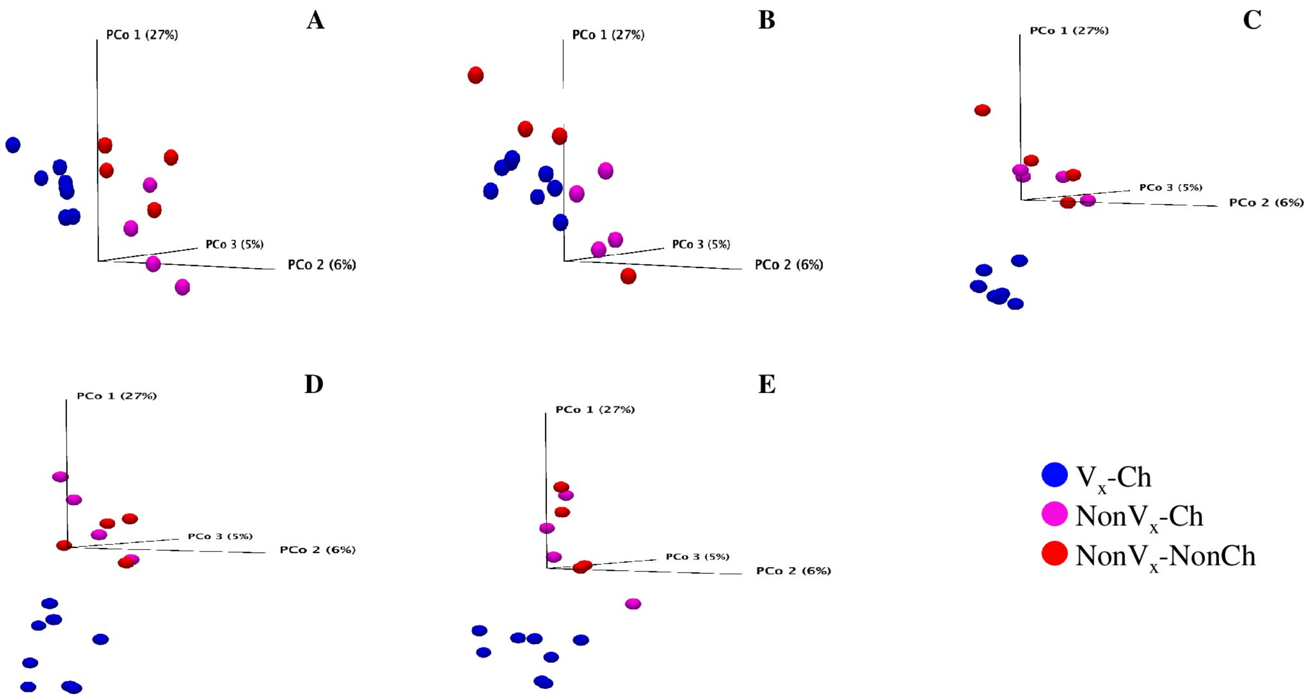

Fig 2. Principal Coordinate Analysis (PCoA) plots comparing bacterial diversity (Beta-diversity) in vaccinated and challenged (Vx-Ch), non-vaccinated and challenged (NonVx-Ch), and non-vaccinated and non-challenged (NonVx-NonCh) calves at weeks, 7 [A], 8 [B], 9 [C], 10 [D], and 11 [E]. Fecal samples on week 7 were collected just before the oral challenge with *E. coli* O157:H7 was given. There was no significant difference in the fecal bacterial community structure between samples from the two vaccinated groups of calves (Vx_E-Ch, Vx_C-Ch) at any time point, therefore the Vx_E-Ch and Vx_C-Ch samples were grouped together (Vx-Ch) for the analysis.

https://doi.org/10.1371/journal.pone.0226099.g002

The analysis and comparisons of alpha-diversity (represented by calculating the Shannon diversity index and Chao 1 estimate of species richness) among the three groups of calves showed that the Shannon diversity index was numerically higher in Vx-Ch group up to week 4 of sampling but this trend was reversed from week 5 and at weeks 10 and 11, and the Shannon diversity index of Vx-Ch calves was significantly (p-value < 0.05) lower than that of NonVx-Ch and NonVx-NonCh groups (**Fig 3A**). Chao 1 species richness was numerically lower in Vx-Ch group for all but weeks 7 and 8 (Fig 3B). Although the difference in Chao 1 species richness was non-significant among three groups up to week 8, there was a significant (p-value $<$ 0.05) reduction in species richness in Vx-Ch group at week 9 (Mean values Vx-Ch vs NonVx-Ch, NonVx-NonCh; 1,022 vs 1,184, 1,185), week 10 (943 vs 1,197, 1,168) and week 11 (1,000 vs 1,255, 1,171) of sampling (**Fig 3B**). The results obtained with Shannon index and species richness corroborate the dynamic changes seen in the community structure (**Fig 2**) wherein the Vx-Ch calves showed a marked change in microbiome after oral challenge with *E. coli* O157:H7. Interestingly, there were no significant differences in the Shannon index or species richness between NonVx-Ch and NonVx-NonCh groups from week 7–11 indicating that oral challenge with *E. coli* O157:H7 alone did not cause significant changes in microbial community structure or alpha-diversity measures.

Microbiomes of *E. coli* O157:H7 vaccinated and challenged calves had differentially abundant genera

The sequencing data generated on Illumina® Miseq was analyzed by MGM 1.6.1 (Qiagen Inc. Redwood City, CA) to create the taxonomic profile of GIT microbiota of all four groups of

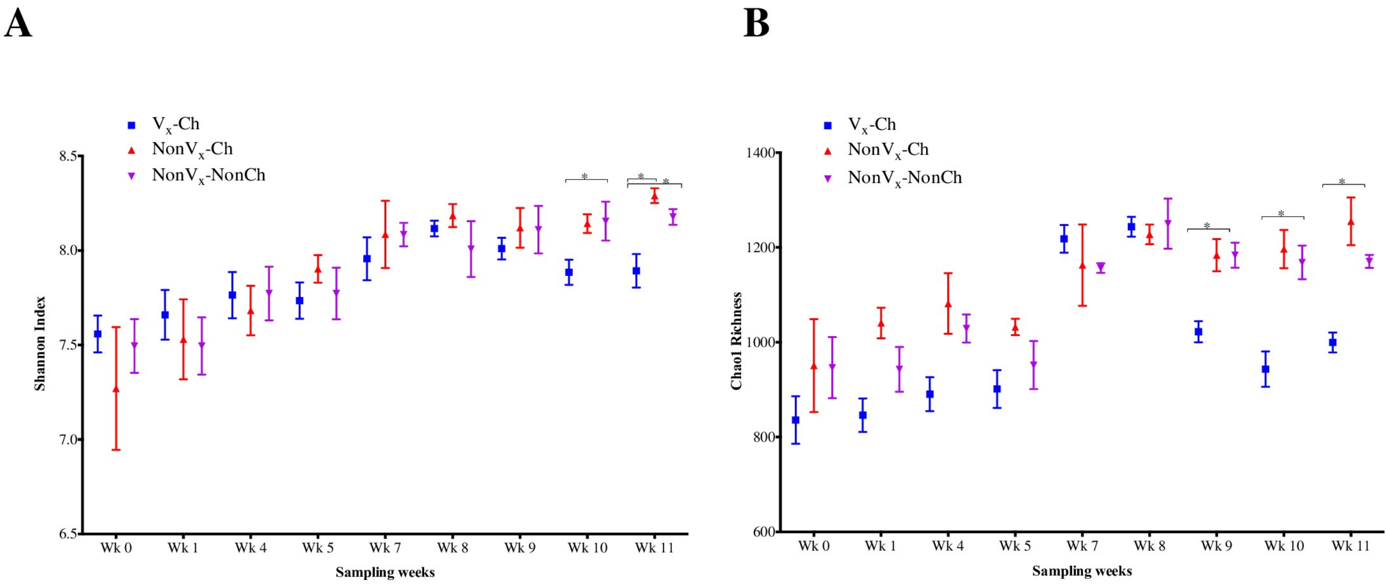

Fig 3. Alpha-diversity represented as scatter plots of the (A) Shannon Index and (B) Chao 1 species richness estimates (mean ± SEM) for three treatment groups: vaccinated and challenged (Vx-Ch), non-vaccinated and challenged (NonVx-Ch), and non-vaccinated and non-challenged (NonVx-NonCh), and for all sampling weeks. There was no significant difference in the fecal bacterial community structure between samples from the two vaccinated groups of calves (Vx_E-Ch, Vx_C-Ch) hence they were grouped together (Vx-Ch) for the analysis. Student's T-test with a threshold p-value < 0.05 was considered significant.

https://doi.org/10.1371/journal.pone.0226099.g003

calves and at all sampling weeks (before and after vaccination and *E. coli* O157:H7 challenge). The type of adjuvant (Emulsigen®-D vs Carbigen™ (MVP Adjuvants™, USA)) used in vaccinating calves did not yield significant differences in the bacterial community structure (p-value > 0.05) and bacterial community structures were not significantly different between vaccinated and non-vaccinated groups at sampling weeks 0, 1 and 4 (**S1 Table**). However, fecal samples collected after the second booster dose of vaccine (at week 5) indicated significant differences in community structure between vaccinated and non-vaccinated groups (Pseudo-f statistic 2.69, p-value = 0.01) (**S1 Table**). We investigated which members of the bacterial community were associated with the significant changes in microbiota due to vaccination at week 5 of sampling. To get the useful information, we selected bacterial genera with overall abundance more than 0.1% so as to avoid bias, sequencing errors, or any genera for which taxonomy was not available. The differential abundance analysis (DAA) of OTU table at the genus level of taxonomy indicated higher relative abundance of *Rikenellaceae* dgA-11 gut group and *Ruminococcaceae* UCG-013 in samples from the vaccinated group and *Anaerorhabdus furcosa* group and *Acetitomaculum* spp. in samples from the non-vaccinated animals (**Fig 4**).

Since oral challenge of calves with *E. coli* O157:H7 alone did not cause significant changes in microbial community structure (**Fig 2**, **S2 Fig**), we compared the taxonomic profiles of GIT microbiota of Vx-Ch and NonVx-Ch groups from week 7 to week 11 by DAA of OTU table at genus level of taxonomy to assess differential impact of vaccination on microbiota composition of these two calf groups. The results of this comparison indicated that a higher number of bacterial genera were differentially abundant between the two groups from week 9 to 11 compared to weeks 7 or 8. These results were in accordance with the observations made about bacterial community structure during these sampling weeks (**Fig 2**). The Vx-Ch calves had higher relative abundance of *Christensenellaceae* R-7 group while *Sutterella* was relatively more abundant in samples from the NonVx-Ch group (**Fig 4**). The DAA analysis at the genus level is more informative compared to the family level because certain members of one bacterial family can be differentially abundant in two groups. For example, *Lachnospiraceae* NK3A20 group

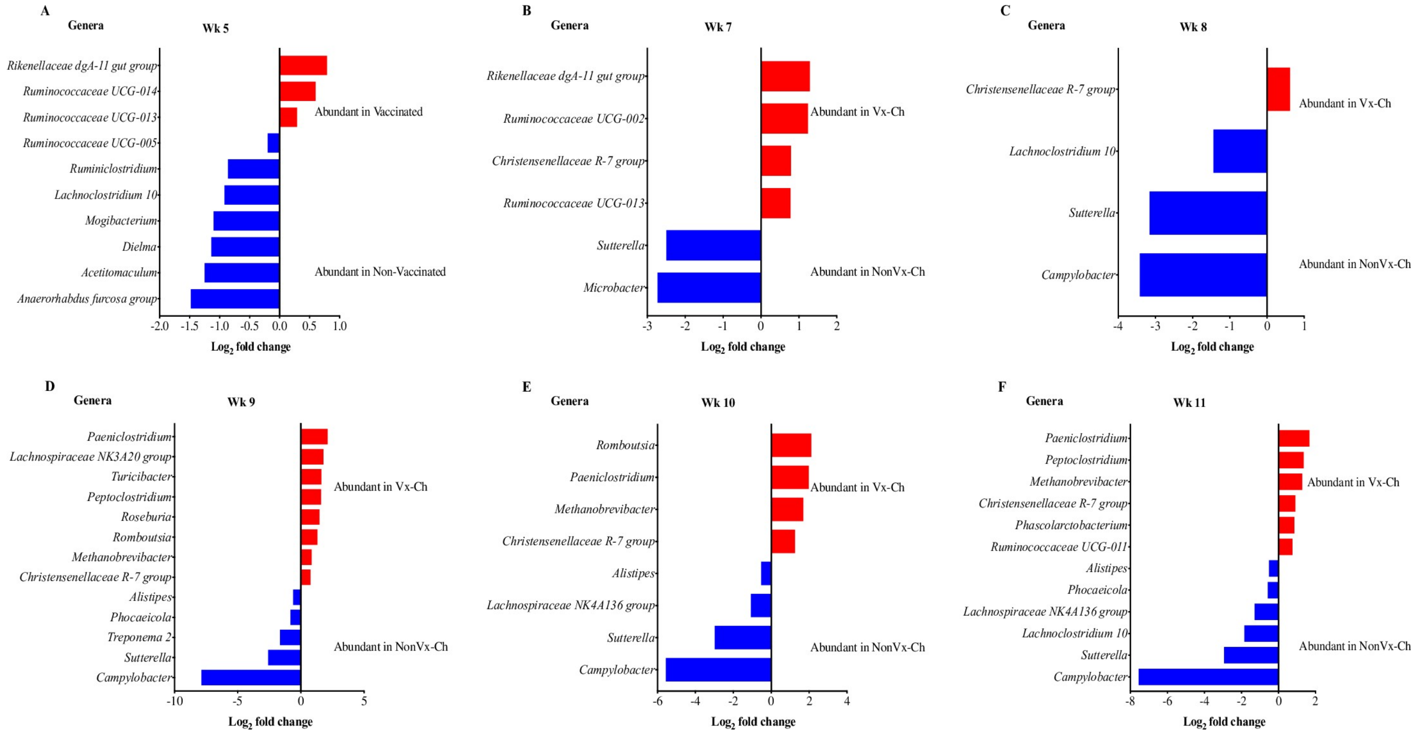

Fig 4. Differential abundance analysis (DAA) of OTUs (genus-level) between (A) all vaccinated and non-vaccinated calves at week 5, and (B-F) all vaccinated and challenged (Vx-Ch), and non-vaccinated and challenged (NonVx-Ch) groups at weeks 7, 8, 9, 10 and 11. The red and blue bars represent genera that are relatively more abundant in the respective group as shown on the plots.

https://doi.org/10.1371/journal.pone.0226099.g004

is relatively more abundant in Vx-Ch calves at week 9, while *Lachnospiraceae* NK4A136 group is relatively more abundant in NonVx-Ch group at week 10 of sampling (**Fig 4**). After the 12 calves (8 calves in Vx-Ch and 4 calves in NonVx-Ch group) were challenged with *E. coli* O157: H7, we observed higher abundance of *Campylobacter* in NonVx-Ch group (from week 8 to 11) while *Romboutsia*, *Paeniclostridium* and *Methanobrevibacter* were more abundant in Vx-Ch group (from week 9 to 11) (**Fig 4**). These results indicated that although there was only a slight change in bacterial community structure due to vaccination, the relative abundance of certain genera was significantly different between the Vx-Ch and the NonVx-Ch calves, after calves received oral challenge of *E. coli* O157:H7.

Vaccination and *E. coli* O157:H7 challenge had no effect on the *Firmicutes*: *Bacteroidetes* (F:B) ratio but altered *Proteobacteria* abundance

We observed a decrease in F:B ratio over the course of this study in all three (Vx-Ch, NonVx-Ch, and NonVx-Non-Ch) calf groups (**Fig 5A**). The Vx-Ch group represents two vaccinated and challenged (Vx_E-Ch and Vx_C-Ch) calf groups. Higher F:B ratio at up to week 5 of sampling could be associated with the housing of these calves allowing pasture grazing during this time. All 16 calves were moved in to a BSL2 building a day before week 7 sampling and were kept there until the end of this study. We did not observe a significant difference in F:B ratio due to vaccination or *E. coli* O157:H7 challenge, except for week 0 sampling (before primary vaccination) when the two vaccinated and challenged (Vx_E-Ch and Vx_C-Ch) calf groups had higher F: B ratio compared to non-vaccinated but challenged (NonVx-Ch) calves (p-value < 0.05, Uncorrected Fisher's Least Significance Difference test for multiple comparisons) (**Fig 5A**).

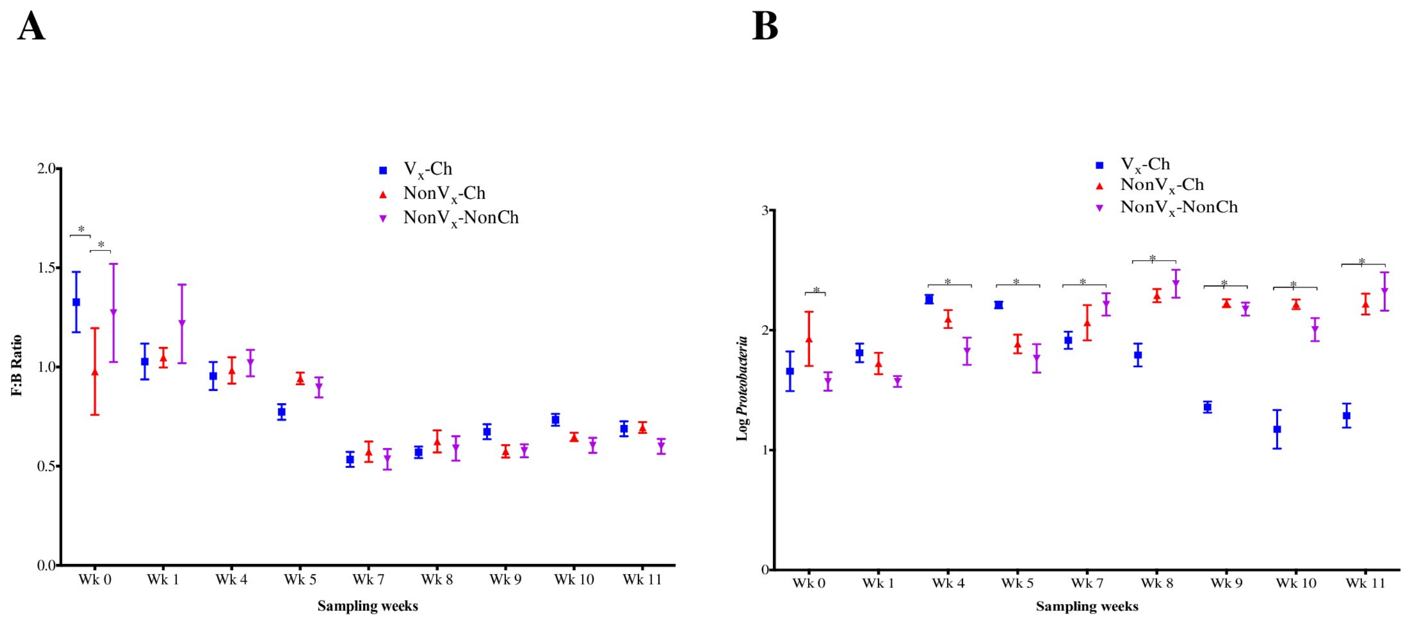

Fig 5. The *Firmicutes*: *Bacteroidetes* (F:B) ratio and the relative abundance of *Proteobacteria* (measured as Log_{10} of number of sequences) between vaccinated and challenged (Vx_Ch), non-vaccinated and challenged (NonVx-Ch), and non-vaccinated and non-challenged (NonVx-NonCh) groups at all sampling weeks. **(A)** F:B ratio: Symbols (one per column) represent mean ± SEM of F:B ratio of all the samples in a group at each sampling week and **(B)** Log_{10} *Proteobacteria*: Symbols (one per column) represent mean ± SEM of relative abundance of *Proteobacteria* (normalized after calculating Log_{10} of number of sequences matching the phylum *Proteobacteria*) of all the samples in a group at each sampling week.

https://doi.org/10.1371/journal.pone.0226099.g005

The relative abundance of members of the phylum *Proteobacteria* (measured as Log_{10} of number of OTUs) showed the effect of both time and vaccination. We observed increase in relative abundance of *Proteobacteria* over the course of study for both NonVx-Ch and NonVx-NonCh calves, but for the Vx-Ch groups of calves, the *Proteobacteria* population relatively increased up to week 5 and then decreased from week 7 to 11 (**Fig 5B**). The abundance of *Proteobacteria* was higher in Vx-Ch compared to NonVx-NonCh calves (significantly higher at weeks 4 (2.26 vs 1.83) and 5 (2.21 vs 1.77) of sampling). However, once the vaccinated animals received oral challenge with *E. coli* O157:H7, the relative abundance of *Proteobacteria* was reduced compared to both NonVx-Ch and NonVx-NonCh calves (**Fig 5B**). We did not observe a significant difference in relative abundance of *Proteobacteria* due to *E. coli* O157:H7 challenge alone, except for week 0 sampling when Vx-Ch calves had higher *Proteobacteria* compared to the NonVx-NonCh group.

E. coli O157:H7 was isolated only from challenged calves

All animals were determined negative for *E. coli* O157:H7 colonization on their arrival as *E. coli* O157:H7 was not cultured from any animal feces prior to challenge. After oral challenge with *E. coli* O157:H7 strain NADC 6564, fecal samples were analyzed for the shedding of the challenge strain. Since we did not observe any significant difference in bacterial community structure between samples from the two vaccinated (Vx_E-Ch and Vx_C-Ch) groups throughout the study, both vaccinated groups (Vx_E-Ch, Vx_C-Ch) were combined in to a single group, called as a vaccinated-challenged group (Vx-Ch), for analysis of the fecal shedding data. All 12 calves in challenged groups (Vx-Ch and NonVx-Ch) shed detectable amounts ($\geq 10^2$ CFU/g feces) of the challenge strain in feces during the course of the study although the concentration (CFU/g feces) was one-log lower in Vx-Ch group of calves compared to the NonVx-Ch calves

on day 43 of week 7 (p-value = 0.046), day 49 of week 8 (p-value = 0.03), and day 56 of week 9 (p-value = 0.03), of sampling (2-way ANOVA, uncorrected Fisher's LSD test) after *E. coli* O157:H7 challenge (S2 Fig).

Vaccination induced significantly high levels of *E. coli* O157:H7-specific serum IgG but not fecal IgA

Vaccine induced *E. coli* O157:H7-specific antibodies in periphery and feces are important indicators of vaccine immunogenicity and are associated with reduction of *E. coli* O157:H7 fecal shedding in vaccinated cattle [10, 13, 14, 30, 34–37]. Immunogenicity can be associated with protection, and given that the vaccine is targeted to an organism that can be commensal, we investigated peripheral *E. coli*-specific IgG and fecal *E. coli*-specific IgA. We first tested sera of four animal groups (Vx_E-Ch, Vx_C-Ch, NonVx-Ch, and NonVx-NonCh) by ELISA to determine differences in the level of induction of *E. coli* O157:H7-specific serum IgG and cross-reactivity of these antibodies to commensal *E. coli*. A preliminary screening of 10-fold serial dilutions (10^{-1} to 10^{-4}) of sera collected from each animal of the four study groups on day 42 of week 7 showed that only sera from the two vaccinated and challenged animal groups (Vx_E-Ch and Vx_C-Ch) had significantly higher reactivity to the vaccine strain (NADC 6597) compared to sera of NonVx-Ch and NonVx-NonCh animals at all tested serum dilutions (S3 Fig). We then tested the pooled sera (Vx-Ch pool containing sera of four Vx_E-Ch and four Vx_C-Ch animals; NonVx-Ch pool containing sera of four NonVx-Ch animals; and NonVx-NonCh pool containing sera of four NonVx-NonCh animals) at 10^{-3} dilution (dilution that produced a strong signal in the preliminary ELISA) (S3 Fig) to determine the specificity of serum IgG response and its cross-reactivity to *E. coli* O157:H7 challenge strain (NADC 6564) and non-pathogenic commensal *E. coli* (NADC 479), respectively. As shown in Fig 6A, the pooled sera of Vx-Ch animal groups showed about 9-fold higher reactivity with *E. coli* O157:H7 challenge strain NADC 6564 (green bars) compared to the commensal strain NADC 479 (red bars). The pooled sera of NonVx-Ch and NonVx-NonCh animals, on the other hand, reacted poorly with

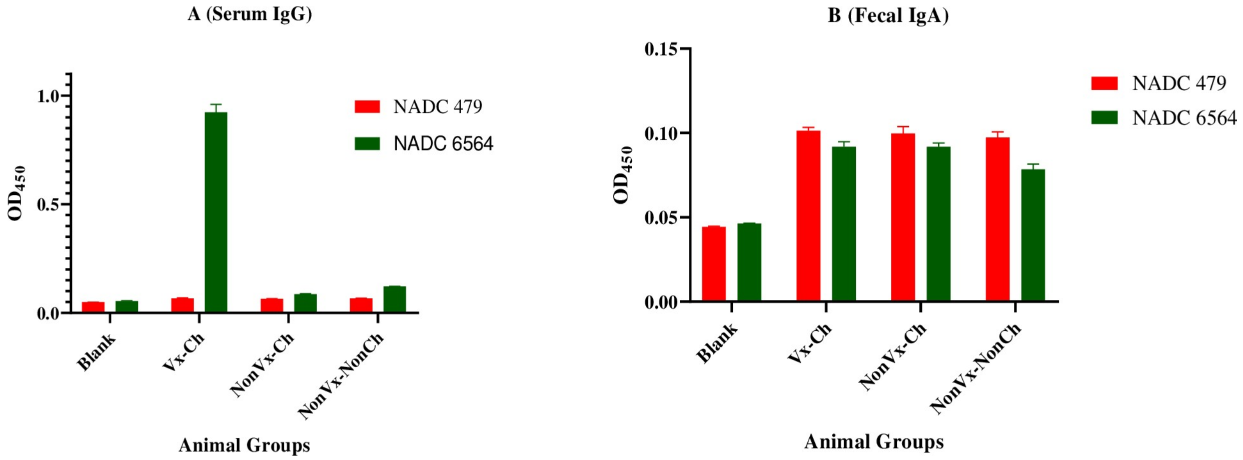

Fig 6. Vaccination induced *E. coli* O157:H7-specific serum IgG but not *E. coli* O157:H7-specifc fecal IgA. (**A**) Relative amounts of serum IgG determined by reacting 10^{-3}-fold dilution of pooled sera representing Vx-Ch, NonVx-Ch, and NonVx-NonCh groups of calves to the commensal *E. coli* NADC 479 (red bar) and the *E. coli* O157:H7 challenge strain NADC 6564 (green bar). (**B**) Bar plot showing specificity of fecal IgA determined by reacting 10-fold diluted, pooled fecal extracts from Vx-Ch, NonVx-Ch, and NonVx-Ch animals to commensal strain NADC 479 (red bars) and *E. coli* O157:H7 challenge strain NADC 6564 (green bars). Serum IgG and fecal IgA levels are represented as Mean ± SD (shown as bars) of three replicate wells of the pooled samples. Statistical analysis was performed using one way-ANOVA with multiple comparison of means. Serum IgG and fecal IgA levels are represented as Mean ± SD (shown as bars) of three replicate wells of the pooled serum or fecal samples. Statistical analysis was performed using one way-ANOVA with multiple comparison of means.

https://doi.org/10.1371/journal.pone.0226099.g006

strain NADC 6564 and commensal strain NADC 479 indicting that the vaccination-induced IgG was specific to *E. coli* O157:H7 and did not cross-react to the nonpathogenic commensal *E. coli* NADC 479. For determining fecal IgA response, we tested pooled fecal extracts (prepared from feces collected immediately before challenge on day 42 of week 7) of vaccinated and non-vaccinated animals (Vx-Ch pool of 4 Vx_E-Ch and 4 Vx_C-Ch animals; NonVc-Ch pool of 4 NonVx-Ch animals; and NonVx-NonCh pool of 4 NonVx-NonCh animals) using a commercially available kit enabling detection of bovine IgA. As shown in Fig 6B, minimal IgA to *E. coli* was detected in the feces, and for the minimal detected, there was no difference in reactivity to *E. coli* O157:H7 (green bars) and generic *E. coli* (red bars).

E. coli O157:H7 vaccination skewed cellular immune responses toward an immunoregulatory phenotype

Previously, vaccination with adjuvanted NADC 6597 induced peripheral *E. coli* O157:H7-specific T cell interferon (IFN)-γ responses, which associated with reduced fecal shedding of *E. coli* O157:H7 from calves [30]. However, in the current study, peripheral *E. coli* O157:H7-specific $CD4^+IFN\text{-}\gamma^+$ cells were not detected after vaccination. Prior to challenge, there were minimal *E. coli* O157:H7-specific T cells responses detected. Primarily $CD4^+$ and $CD8^+$ cells responded to *E. coli* O157:H7-restimulation with the production of IL-10, but not IFN-γ (Fig 7). The

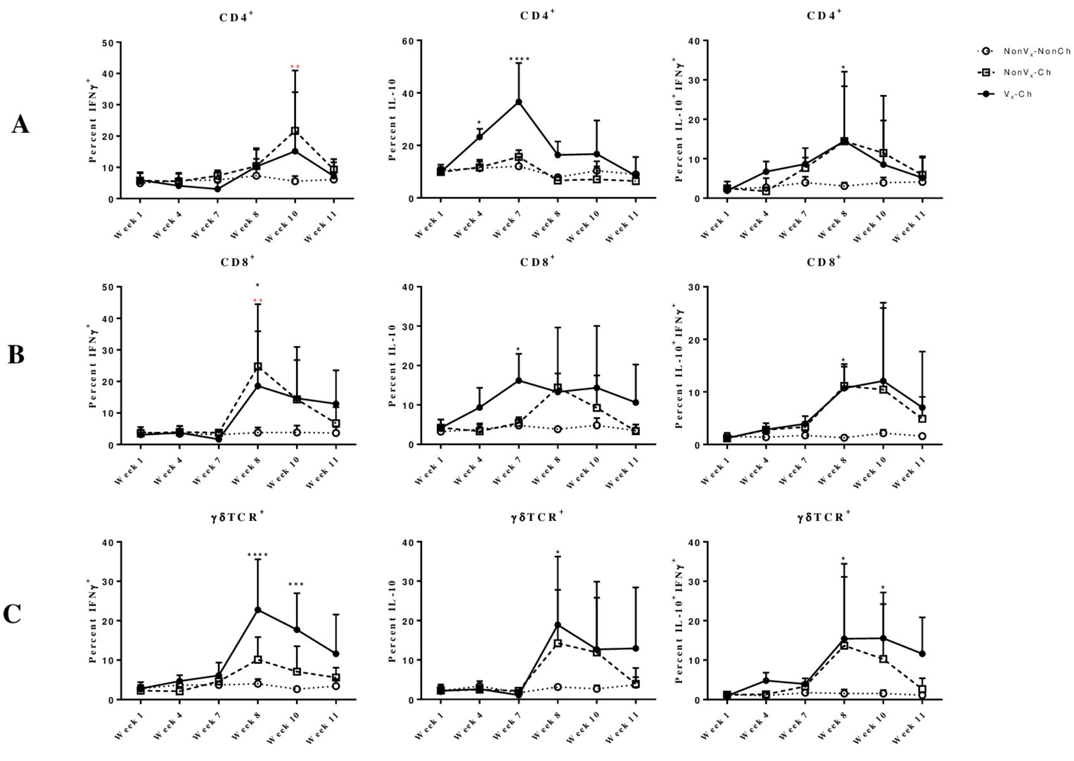

Fig 7. Percentage of IL-10 producing cells was greater than IFN-γ producing cells in vaccinated and challenged animals. Peripheral T cells were assessed for intracellular IFN-γ and IL-10 after 3-day stimulation of PBMC with *E. coli* O157:H7 antigen *ex vivo*. Panel A represents $CD4^+$ lymphocytes; Panel B represents $CD8^+$ lymphocytes, and Panel C represents $\gamma\delta TCR^+$ lymphocytes. Graphs in the left, middle, and right panels represent cells positive for intracellular IFN-γ, intracellular IL-10, or both intracellular IFN-γ and IL-10, respectively. Treatment groups are shown as open circles (NonVx-NonCh), boxes (NonVx-Ch), and filled circles (Vx-Ch). Black and red statistical symbols (asterisks) are comparisons between Vx-Ch and NonVx-NonCh groups, and NonVx-Ch and NonVx-NonCh groups, respectively. Bars represent Mean ± SD. n = 4 for NonVx-NonCh and NonVx-Ch and n = 8 for Vx-Ch. Statistical analysis was performed utilizing two way-ANOVA with parameters for time and experimental group with multiple comparison of means.

https://doi.org/10.1371/journal.pone.0226099.g007

percentage of $CD4^{+}IL\text{-}10^{+}$ cells was significantly greater at week 4 and 7 in Vx-Ch calves compared to NonVx-Ch and NonVx-NonCh groups ($p<0.05$, $p<0.0001$ respectively) (**Fig 7, Panel A**). The percentage of $CD8^{+}$ $IL\text{-}10^{+}$ was significantly greater at week 7 in Vx-Ch and NonVx-Ch calves compared to NonVx-NonCh calves ($p<0.05$) (**Fig 7, Panel B**).

Challenge, but not prior vaccination, impacted peripheral *E. coli* O157:H7-specific γδ T cell responses, as percentages of cytokine producing cells were similar between Vx-Ch and NonVx-Ch compared to NonVx-NonCh group. Specifically, the percentage of $CD4^{+}$ cells producing IFN-γ^{+} after *in vitro* stimulation with *E. coli* O157:H7 antigen was significantly greater in Vx-Ch and NonVx-Ch calf groups compared to the NonVx-NonCh group (week 10, $p<0.01$) (**Fig 7, Panel A**). Following challenge, the percentage of $CD4^{+}IFN\text{-}\gamma^{+}IL\text{-}10^{+}$ cells was significantly greater in Vx-Ch and NonVx-Ch calves compared to the NonVx-NonCh calves (week 8, $p<0.05$) (**Fig 7, Panel A**). The percentage of $CD8^{+}IFN\text{-}\gamma^{+}$ was significantly greater at week 8 in NonVx-Ch ($p<0.01$) and Vx-Ch calves ($p<0.05$) compared to the NonVx-Non-Ch animals (**Fig 7, Panel B**). The percentage of $CD4^{+}IFN\text{-}\gamma^{+}IL\text{-}10^{+}$ cells was significantly greater at week 8 for the Vx-Ch and NonVx-Ch groups of calves compared to NonVx-NonCh calves ($p<0.05$) (**Fig 7, Panel B**).

Unlike γδ T cells, the percentage of γδ T cells producing IFN-γ upon *in vitro* restimulation were impacted by prior vaccination. The percentage of $\gamma\delta TCR^{+}$ intracellular IFN-γ was significantly greater in Vx-Ch calves at week 8 ($p < 0.0001$) and week 10 ($p < 0.001$) compared to NonVx-Ch and NonVx-NonCh calves (**Fig 7, Panel C**). However, the percentage of $\gamma\delta TCR^{+}IL\text{-}10^{+}$ cells was significantly greater at week 8 in both Vx-Ch and NonVx-Ch calves ($p<0.05$) compared to NonVx-NonCh calves (**Fig 7, Panel C**). Overall, IL-10 producing cells were prominent both before challenge and after, and vaccination only impacted IFN-γ producing γδ T cells, but only after challenge.

Discussion

Shiga toxin producing *E. coli* (STEC) cause 2.8 million illnesses globally [38], more than 265,000 illnesses in the US [39] and 36% of these illnesses are attributed to *E. coli* O157:H7 alone. In addition, a combined economic loss of about $993 million per year to public health, agriculture and meat industry has been attributed to STEC (mainly *E. coli* O157:H7) contamination and infections [40, 41]. Cattle vaccination presents a viable pre-harvest strategy to reduce contamination and human infections as cattle are the primary *E. coli* O157:H7 reservoirs [7]. The role of the gastrointestinal (GIT) microbiota and bacterially-derived products in modulating local intestinal and systemic immune responses are well established [42]. Unfortunately, *E. coli* O157:H7 can be a commensal organism such as in cattle or a pathogen when it infects humans. Vaccination itself may alter the GIT microbiota, and would be important to understand. Vaccine formulations that do not adversely affect or do constructively affect GIT microbiota may minimize other impacts on animal health. In the current study, we evaluated the effect of vaccination and *E. coli* O157:H7 challenge on the GIT bacterial community structure of calves and immunogenicity of the vaccine formulation immune responses targeting *E. coli* O157:H7.

Vaccination and *E. coli* O157:H7 challenge altered the fecal microbiota but the two vaccine formulations used did not cause changes in the fecal microbiota. Thus, data from the two vaccinated groups were pooled for the analyses. Genera like *Paeniclostridium* and *Chritensenellaceae* R-7 group were relatively more abundant in GIT microbial communities of calves vaccinated and challenged (Vx-Ch) with *E. coli* O157:H7. At week 7 (on or before day 42) of sampling, which was prior to challenge, a higher relative abundance of *Rikenellaceae* dgA-11 gut group was detected in vaccinated calves compared to non-vaccinated calf groups.

Following challenge (week 10 of study) a higher relative abundance of *Romboutsia* and *Paeniclostridium* was detected in vaccinated (Vx-Ch) calves while challenged non-vaccinated calves (NonVx-Ch) had higher abundance of *Sutterella* and *Campylobacter*. Thus, vaccination alone did shift microbial communities, and challenge also had an impact.

The role and impact of GIT microbiota composition in domestic animals in bacterial colonization/pathogenesis is largely unknown, as evidenced by the relatively small number of studies compared to the ones evaluating the human microbiome [18, 25]. We have identified several differentially abundant bacterial genera (*Romboutsia*, *Paeniclostridium*, *Methanobrevibacter*, and *Turicibacter*) associated with vaccination which will be useful in future studies for identifying association of microbiota with vaccination in cattle. Specifically, we observed higher relative abundance of *Turicibacter* in Vx-Ch calves at week 9 (**Fig 4**) without noticing any significant effect on F:B ratio. Previous studies have demonstrated higher abundance of *Turicibacter* in the GIT of cattle that were fed feedlot ration and had higher F:B ratio [18, 20]. *Turicibacter* are reportedly also enriched in the large intestine of cattle which contained relatively lower abundance of *Proteobacteria* [17]. We also consistently observed higher abundance of *Sutterella* and *Campylobacter* in NonVx-Ch calves. Further studies involving cattle are needed to determine the functional profile of the GIT microbiota due to vaccination.

Oral challenge with *E. coli* O157:H7 by itself did not cause significant changes in microbial community structure. Change in the bacterial community structure was significantly associated with vaccination but independent of the adjuvants used in the vaccine formulations. The significant differences in the GIT bacterial community structure and taxonomic profiles were between Vx-Ch and NonVx-Ch calves, but we did not follow these animals for an extended period to see if the GIT microbiota of Vx-Ch animals would become similar to that of the NonVx-Ch animals over time. A slight reduction in the F:B ratio was observed over the course of the study, regardless of the vaccination/challenge status of calves. However, the abundance of *Proteobacteria* was significantly greater before and lower after challenge with *E. coli* O157:H7 in the vaccinated group (Vx-Ch) compared to the non-vaccinated but challenged group (NonVx-Ch).

A variety of vaccine candidates including subunit vaccines prepared from specific purified *E. coli* O157:H7 proteins, SRP vaccine based on siderophore receptor and porin proteins of *E. coli* O157:H7, culture supernatants containing type III-secreted proteins of *E. coli* O157:H7, or bacterial ghosts of *E. coli* O157:H7 have shown induction of *E. coli* O157:H7-specific IgG, IgA, or both IgG and IgA [9, 10, 14, 30, 35, 36, 43]. In majority of the vaccination studies conducted using above mentioned vaccines, serum *E. coli* O157:H7-specific IgG appeared to be the major circulating immune response generated by vaccinated animals that also showed reduction in the fecal shedding of experimental *E. coli* O157:H7 challenge strain or naturally infecting strain of *E. coli* O157:H7. In a previous study, we demonstrated the induction of both *E. coli* O157:H7-specific serum IgG and fecal IgA in animals vaccinated with a vaccine formulation containing the inactivated vaccine strain (*E. coli* O157:H7 NADC 6597) and ISA61 Montenide adjuvant [14]. The *E. coli* O157:H7-specific IgA exerted inhibitory effect on the adherence of *E. coli* O157:H7 to cultured epithelial cells, thus demonstrating functional specificity of this IgA against *E. coli* O157:H7. However, in the current study examining the effects of vaccination on intestinal microbiota diversity, we observed that the two vaccine formulations prepared by mixing the inactivated *E. coli* O157:H7 strain NADC 6597 with adjuvant Emulsigen-D or Carbigen only induced higher levels of *E. coli* O157:H7-specific IgG but not *E. coli* O157:H7-specific fecal IgA. It could be hypothesized that the increases in *E. coli* O157:H7-specific IgG might be important considering that after oral challenge with *E. coli* O157:H7 strain NADC 6564, all 12 calves in the three challenged groups (Vx_D-Ch, Vx_C-Ch, and NonVx-Ch group) continued to shed the challenge strain in feces, but the amount (CFU/g feces) of *E. coli* O157:H7 shed in feces was one-log lower on week 7 (day 43), week 8 (day 49), and week 9 (day

56) in Vx-Ch group (representing both Vx_D-Ch, Vx_C-Ch groups) of calves compared to the NonVx-Ch calves. Several studies have reported that the circulating IgG could cross translum-inal barriers to reach intestinal mucosal surfaces where these could interact with specific path-ogenic entities and provide protection against the targeted pathogen [44]. Since we observed significant differences in the GIT bacterial community structure and taxonomic profiles between Vx-Ch and NonVx-Ch calves after week seven, when IgG response reached the high-est concentration in Vx-Ch animals, the enhanced levels of serum IgG could have directly or indirectly influence fecal shedding of *E. coli* O157:H7 and intestinal microbial community structure and diversity.

In conclusion, we have demonstrated that vaccination altered the composition of the GIT microbial population community structure and subsequent experimental exposure of vacci-nated animals to *E. coli* O157:H7 resulted in additional changes in the GIT microbiota com-munity structure that were not seen in the unvaccinated but *E. coli* O157:H7-exposed animals. Since vaccination did transiently reduce shedding of *E. coli* O157:H7 for three weeks post-*E. coli* O157:H7 exposure of vaccinated animals, the microbiota community structure could be considered constructive but suboptimal in their ability to repress fecal shedding in vaccinated animals for longer time periods. It is also possible that besides inducing specific microbiota shifts, vaccination must also induce both *E. coli* O157:H7-sepcific antibody and cell-mediated immune responses in order to provide optimal protection in terms of reducing the magnitude of *E. coli* O157:H7 fecal shedding for longer time periods. In the current study, we were able to show that the vaccine formulations that we used had no adverse vaccine site reactivity in ani-mals, were able to induce significantly high levels of peripheral IgG response, but generated no fecal IgA response, did not induce peripheral *E. coli* O157:H7-specific $CD4^+$ IFN-γ^+ cells, and produced IL-10 upon restimulation but no IFN-γ. In a previous study, we demonstrated that a vaccine formulation formulated with the same vaccine strain that was used in the current study but mixed with a different adjuvant induced significantly high levels of *E. coli* O157:H7-specific peripheral IgG, fecal IgA, cell-mediated immune cells ($CD4^+$ IFN-γ^+ cells), and immune markers (IFN-γ^+) and these vaccinated animals also showed significant reduction in fecal shedding over the duration of the study [14]. To strengthen the findings of the study described in the current manuscript, future studies are planned to investigate changes in the bovine GIT microbiota over an extended period of time with optimal vaccine formulations and to determine the effects of vaccine-induced immune responses and altered microbiota in limiting/enhancing immune responses to *E. coli* O157:H7.

Supporting information

S1 Table. Differences in bacterial community structure between the different treatment groups. Differences in bacterial community structure were examined by PERMANOVA anal-ysis of the bacterial community structure (Beta-diversity).
(DOCX)

S1 Fig. Principal coordinate analysis (PCoA, Beta-diversity) plots. PCoA was performed for comparing bacterial community structure between vaccinated and non-vaccinated groups at sampling weeks 0, 1, 4 and 5, before the *E. coli* O157:H7 challenge was given.
(TIF)

S2 Fig. Fecal shedding of *E. coli* O157:H7 before and after experimental inoculation. Num-ber of bacteria shed was first Log transformed (represented as Log_{10} CFU/g feces) and com-pared by ANOVA for difference between groups over a period of 12 weeks of sampling.
(TIF)

S3 Fig. Vaccination of calves induced *E. coli* O157:H7-specific serum IgG. The serum IgG induced after vaccination was determined by reacting 10-fold serial dilutions of serum of each of the four calves from Vx_E-Ch, Vx_C-Ch, NonVx-Ch, and pooled sera of the four calves of NonVx-NonCh groups to the *E. coli* O157:H7 vaccine strain NADC 6597. Serum IgG levels are represented as Mean ± SD (shown as bars) of three replicate wells of the serum samples. Statistical analysis was performed using one way-ANOVA with multiple comparison of means.
(TIF)

Acknowledgments

Technical support provided by Bryan Wheeler, Lindsay Andersen, Brian Conrad, David Alt, Jennifer Jones, and Julian Trachsel with sample collection and DNA sequencing is appreciated. This work was supported by USDA-ARS CRIS projects 5030-32000-112-00D and 5030-32000-115-00D. Mention of trade names or commercial products in this article is solely for the purpose of providing specific information and does not imply recommendation or endorsement by the U.S. Department of Agriculture. USDA is an equal opportunity provider and employer.

Author Contributions

Conceptualization: Raies A. Mir, Indira T. Kudva, Vijay K. Sharma.

Data curation: Raies A. Mir, Robert G. Schaut, Indira T. Kudva, Vijay K. Sharma.

Formal analysis: Raies A. Mir, Robert G. Schaut, Indira T. Kudva, Vijay K. Sharma.

Methodology: Raies A. Mir, Robert G. Schaut, Torey Looft, Indira T. Kudva, Vijay K. Sharma.

Project administration: Indira T. Kudva, Vijay K. Sharma.

Resources: Heather K. Allen, Indira T. Kudva, Vijay K. Sharma.

Software: Raies A. Mir, Robert G. Schaut, Indira T. Kudva.

Supervision: Indira T. Kudva, Vijay K. Sharma.

Validation: Raies A. Mir, Robert G. Schaut, Indira T. Kudva, Vijay K. Sharma.

Visualization: Raies A. Mir, Robert G. Schaut, Indira T. Kudva, Vijay K. Sharma.

Writing – original draft: Raies A. Mir, Robert G. Schaut, Indira T. Kudva, Vijay K. Sharma.

Writing – review & editing: Raies A. Mir, Robert G. Schaut, Heather K. Allen, Torey Looft, Crystal L. Loving, Indira T. Kudva, Vijay K. Sharma.

References

1. Davis MA, Cloud-Hansen KA, Carpenter J, Hovde CJ. *Escherichia coli* O157:H7 in environments of culture-positive cattle. Appl Environ Microbiol. 2005; 71(11):6816–22. Epub 2005/11/05. https://doi.org/10.1128/AEM.71.11.6816-6822.2005 PMID: 16269714; PubMed Central PMCID: PMC1287631.
2. Naylor SW, Low JC, Besser TE, Mahajan A, Gunn GJ, Pearce MC, et al. Lymphoid Follicle-Dense Mucosa at the Terminal Rectum Is the Principal Site of Colonization of Enterohemorrhagic *Escherichia coli* O157:H7 in the Bovine Host. Infection and Immunity. 2003; 71(3):1505–12. https://doi.org/10.1128/IAI.71.3.1505-1512.2003 PMID: 12595469
3. Pruimboom-Brees IM, Morgan TW, Ackermann MR, Nystrom ED, Samuel JE, Cornick NA, et al. Cattle lack vascular receptors for *Escherichia coli* O157:H7 Shiga toxins. Proc Natl Acad Sci U S A. 2000; 97(19):10325–9. Epub 2000/09/06. https://doi.org/10.1073/pnas.190329997 PMID: 10973498; PubMed Central PMCID: PMC27023.

4. Davis TK, Van De Kar NC, Tarr PI. Shiga Toxin/Verocytotoxin-Producing *Escherichia coli* Infections: Practical Clinical Perspectives. Microbiol Spectr. 2014; 2(4):EHEC-0025-2014. Epub 2015/06/25. https://doi.org/10.1128/microbiolspec.EHEC-0025-2014 PMID: 26104210.
5. Riley LW, Remis RS, Helgerson SD, McGee HB, Wells JG, Davis BR, et al. Hemorrhagic colitis associated with a rare *Escherichia coli* serotype. N Engl J Med. 1983; 308(12):681–5. Epub 1983/03/24. https://doi.org/10.1056/NEJM198303243081203 PMID: 6338386.
6. Elder RO, Keen JE, Siragusa GR, Barkocy-Gallagher GA, Koohmaraie M, Laegreid WW. Correlation of enterohemorrhagic *Escherichia coli* O157 prevalence in feces, hides, and carcasses of beef cattle during processing. Proc Natl Acad Sci USA. 2000; 97(7):5.
7. Matthews L, Reeve R, Gally DL, Low JC, Woolhouse ME, McAteer SP, et al. Predicting the public health benefit of vaccinating cattle against *Escherichia coli* O157. Proc Natl Acad Sci U S A. 2013; 110 (40):16265–70. Epub 2013/09/18. https://doi.org/10.1073/pnas.1304978110 PMID: 24043803; PubMed Central PMCID: PMC3791763.
8. Varela NP, Dick P, Wilson J. Assessing the Existing Information on the Efficacy of Bovine Vaccination against *Escherichia coli* O157:H7 A Systematic Review and Meta-analysis. Zoonoses Public Hlth. 2013; 60(4):253–68. https://doi.org/10.1111/j.1863-2378.2012.01523.x WOS:000318098500001. PMID: 22856462
9. Fox JT, Thomson DU, Drouillard JS, Thornton AB, Burkhardt DT, Emery DA, et al. Efficacy of *Escherichia coli* O157:H7 siderophore receptor/porin proteins-based vaccine in feedlot cattle naturally shedding E. coli O157. Foodborne Pathog Dis. 2009; 6(7):893–9. Epub 2009/09/10. https://doi.org/10.1089/fpd.2009.0336 PMID: 19737065.
10. Potter AA, Klashinsky S, Li Y, Frey E, Townsend H, Rogan D, et al. Decreased shedding of *Escherichia coli* O157:H7 by cattle following vaccination with type III secreted proteins. Vaccine. 2004; 22(3–4):362–9. Epub 2003/12/13. https://doi.org/10.1016/j.vaccine.2003.08.007 PMID: 14670317.
11. Stanford K, Hannon S, Booker CW, Jim GK. Variable efficacy of a vaccine and direct-fed microbial for controlling *Escherichia coli* O157:H7 in feces and on hides of feedlot cattle. Foodborne Pathog Dis. 2014; 11(5):379–87. Epub 2014/03/29. https://doi.org/10.1089/fpd.2013.1693 PMID: 24673729.
12. Thomson DU, Loneragan GH, Thornton AB, Lechtenberg KF, Emery DA, Burkhardt DT, et al. Use of a siderophore receptor and porin proteins-based vaccine to control the burden of *Escherichia coli* O157:H7 in feedlot cattle. Foodborne Pathog Dis. 2009; 6(7):871–7. Epub 2009/09/10. https://doi.org/10.1089/fpd.2009.0290 PMID: 19737063.
13. Sharma VK, Dean-Nystrom EA, Casey TA. Evaluation of *hha* and *hha sepB* mutant strains of *Escherichia coli* O157:H7 as bacterins for reducing E. coli O157:H7 shedding in cattle. Vaccine. 2011; 29 (31):5078–86. Epub 2011/05/10. https://doi.org/10.1016/j.vaccine.2011.04.073 PMID: 21550373.
14. Sharma VK, Schaut RG, Loving CL. Vaccination with killed whole-cells of *Escherichia coli* O157:H7 *hha* mutant emulsified with an adjuvant induced vaccine strain-specific serum antibodies and reduced *E. coli* O157:H7 fecal shedding in cattle. Vet Microbiol. 2018; 219:190–9. Epub 2018/05/21. https://doi.org/10.1016/j.vetmic.2018.04.003 PMID: 29778196.
15. Nguyen QN, Himes JE, Martinez DR, Permar SR. The Impact of the Gut Microbiota on Humoral Immunity to Pathogens and Vaccination in Early Infancy. PLoS Pathog. 2016; 12(12):e1005997. Epub 2016/12/23. https://doi.org/10.1371/journal.ppat.1005997 PMID: 28006021; PubMed Central PMCID: PMC5179050.
16. Oh JZ, Ravindran R, Chassaing B, Carvalho FA, Maddur MS, Bower M, et al. TLR5-mediated sensing of gut microbiota is necessary for antibody responses to seasonal influenza vaccination. Immunity. 2014; 41(3):478–92. Epub 2014/09/16. https://doi.org/10.1016/j.immuni.2014.08.009 PMID: 25220212; PubMed Central PMCID: PMC4169736.
17. Mao S, Zhang M, Liu J, Zhu W. Characterising the bacterial microbiota across the gastrointestinal tracts of dairy cattle: membership and potential function. Sci Rep. 2015; 5:16116. Epub 2015/11/04. https://doi.org/10.1038/srep16116 PMID: 26527325; PubMed Central PMCID: PMC4630781.
18. Callaway TR, Dowd SE, Edrington TS, Anderson RC, Krueger N, Bauer N, et al. Evaluation of bacterial diversity in the rumen and feces of cattle fed different levels of dried distillers grains plus solubles using bacterial tag-encoded FLX amplicon pyrosequencing. J Anim Sci. 2010; 88(12):3977–83. Epub 2010/08/24. https://doi.org/10.2527/jas.2010-2900 PMID: 20729286.
19. Thomas M, Webb M, Ghimire S, Blair A, Olson K, Fenske GJ, et al. Metagenomic characterization of the effect of feed additives on the gut microbiome and antibiotic resistome of feedlot cattle. Sci Rep. 2017; 7(1):12257. Epub 2017/09/28. https://doi.org/10.1038/s41598-017-12481-6 PMID: 28947833; PubMed Central PMCID: PMC5612972.
20. Myer PR, Freetly HC, Wells JE, Smith TPL, Kuehn LA. Analysis of the gut bacterial communities in beef cattle and their association with feed intake, growth, and efficiency. Journal of Animal Science. 2017; 95 (7). https://doi.org/10.2527/jas2016.1059

21. Petri RM, Schwaiger T, Penner GB, Beauchemin KA, Forster RJ, McKinnon JJ, et al. Characterization of the core rumen microbiome in cattle during transition from forage to concentrate as well as during and after an acidotic challenge. PLoS One. 2013; 8(12):e83424. Epub 2014/01/07. https://doi.org/10.1371/journal.pone.0083424 PMID: 24391765; PubMed Central PMCID: PMC3877040.

22. Benckert J, Schmolka N, Kreschel C, Zoller MJ, Sturm A, Wiedenmann B, et al. The majority of intestinal IgA+ and IgG+ plasmablasts in the human gut are antigen-specific. J Clin Invest. 2011; 121 (5):1946–55. Epub 2011/04/15. https://doi.org/10.1172/JCI44447 PMID: 21490392; PubMed Central PMCID: PMC3083800.

23. Atarashi K, Tanoue T, Shima T, Imaoka A, Kuwahara T, Momose Y, et al. Induction of Colonic Regulatory T Cells by Indigenous Clostridium Species. Science. 2011; 331:5. https://doi.org/10.1126/science.1198469 PMID: 21205640

24. Jami E, White BA, Mizrahi I. Potential role of the bovine rumen microbiome in modulating milk composition and feed efficiency. PLoS One. 2014; 9(1):e85423. Epub 2014/01/28. https://doi.org/10.1371/journal.pone.0085423 PMID: 24465556; PubMed Central PMCID: PMC3899005.

25. Li Q, Lauber CL, Czarnecki-Maulden G, Pan Y, Hannah SS. Effects of the Dietary Protein and Carbohydrate Ratio on Gut Microbiomes in Dogs of Different Body Conditions. MBio. 2017; 8(1). Epub 2017/01/26. https://doi.org/10.1128/mBio.01703-16 PMID: 28119466; PubMed Central PMCID: PMC5263242.

26. Sampson TR, Debelius JW, Thron T, Janssen S, Shastri GG, Ilhan ZE, et al. Gut Microbiota Regulate Motor Deficits and Neuroinflammation in a Model of Parkinson's Disease. Cell. 2016; 167(6):1469–80 e12. Epub 2016/12/03. https://doi.org/10.1016/j.cell.2016.11.018 PMID: 27912057; PubMed Central PMCID: PMC5718049.

27. Lima FS, Oikonomou G, Lima SF, Bicalho ML, Ganda EK, Filho JC, et al. Prepartum and postpartum rumen fluid microbiomes: characterization and correlation with production traits in dairy cows. Appl Environ Microbiol. 2015; 81(4):1327–37. Epub 2014/12/17. https://doi.org/10.1128/AEM.03138-14 PMID: 25501481; PubMed Central PMCID: PMC4309715.

28. Sharma VK, Bayles DO, Alt DP, Looft T. Complete Genome Sequences of Curli-Negative and Curli-Positive Isolates of Foodborne *Escherichia coli* O157:H7 Strain 86–24. Genome Announc. 2016; 4(6). Epub 2016/12/17. https://doi.org/10.1128/genomeA.01323-16 PMID: 27979932; PubMed Central PMCID: PMC5159565.

29. Sharma VK, Zuerner RL. Role of *hha* and *ler* in transcriptional regulation of the *esp* operon of enterohemorrhagic *Escherichia coli* O157:H7. J Bacteriol. 2004; 186(21):7290–301. Epub 2004/10/19. https://doi.org/10.1128/JB.186.21.7290-7301.2004 PMID: 15489441; PubMed Central PMCID: PMC523200.

30. Schaut RG, Boggiatto PM, Loving CL, Sharma VK. Cellular and Mucosal Immune Responses Following Vaccination with Inactivated Mutant of *Escherichia coli* O157:H7. Sci Rep. 2019; 9(1):6401. Epub 2019/04/27. https://doi.org/10.1038/s41598-019-42861-z PMID: 31024031; PubMed Central PMCID: PMC6483982.

31. Kozich JJ, Westcott SL, Baxter NT, Highlander SK, Schloss PD. Development of a dual-index sequencing strategy and curation pipeline for analyzing amplicon sequence data on the MiSeq Illumina sequencing platform. Appl Environ Microbiol. 2013; 79(17):5112–20. Epub 2013/06/25. https://doi.org/10.1128/AEM.01043-13 PMID: 23793624; PubMed Central PMCID: PMC3753973.

32. Allen HK, Bayles DO, Looft T, Trachsel J, Bass BE, Alt DP, et al. Pipeline for amplifying and analyzing amplicons of the V1-V3 region of the 16S rRNA gene. BMC Res Notes. 2016; 9:380. Epub 2016/08/04. https://doi.org/10.1186/s13104-016-2172-6 PMID: 27485508; PubMed Central PMCID: PMC4970291.

33. Quast C, Pruesse E, Yilmaz P, Gerken J, Schweer T, Yarza P, et al. The SILVA ribosomal RNA gene database project: improved data processing and web-based tools. Nucleic Acids Res. 2013; 41(Database issue):D590–6. Epub 2012/11/30. https://doi.org/10.1093/nar/gks1219 PMID: 23193283; PubMed Central PMCID: PMC3531112.

34. McNeilly TN, Mitchell MC, Nisbet AJ, McAteer S, Erridge C, Inglis NF, et al. IgA and IgG antibody responses following systemic immunization of cattle with native H7 flagellin differ in epitope recognition and capacity to neutralise TLR5 signalling. Vaccine. 2010; 28(5):1412–21. Epub 2009/11/21. https://doi.org/10.1016/j.vaccine.2009.10.148 PMID: 19925908.

35. McNeilly TN, Naylor SW, Mahajan A, Mitchell MC, McAteer S, Deane D, et al. *Escherichia coli* O157:H7 colonization in cattle following systemic and mucosal immunization with purified H7 flagellin. Infect Immun. 2008; 76(6):2594–602. Epub 2008/03/26. https://doi.org/10.1128/IAI.01452-07 PMID: 18362130; PubMed Central PMCID: PMC2423056.

36. Thornton AB, Thomson DU, Loneragan GH, Fox JT, Burkhardt DT, Emery DA, et al. Effects of a siderophore receptor and porin proteins-based vaccination on fecal shedding of *Escherichia coli* O157:H7 in experimentally inoculated cattle. J Food Prot. 2009; 72(4):866–9. Epub 2009/05/14. https://doi.org/10.4315/0362-028x-72.4.866 PMID: 19435240.

37. Vilte DA, Larzabal M, Garbaccio S, Gammella M, Rabinovitz BC, Elizondo AM, et al. Reduced faecal shedding of *Escherichia coli* O157:H7 in cattle following systemic vaccination with gamma-intimin C(2)(8)(0) and EspB proteins. Vaccine. 2011; 29(23):3962–8. Epub 2011/04/12. https://doi.org/10.1016/j.vaccine.2011.03.079 PMID: 21477674.

38. Majowicz SE, Scallan E, Jones-Bitton A, Sargeant JM, Stapleton J, Angulo FJ, et al. Global incidence of human Shiga toxin-producing *Escherichia coli* infections and deaths: a systematic review and knowledge synthesis. Foodborne Pathog Dis. 2014; 11(6):447–55. Epub 2014/04/23. https://doi.org/10.1089/fpd.2013.1704 PMID: 24750096; PubMed Central PMCID: PMC4607253.

39. Scallan EG, Patricia M. A, Frederick J. T, V. R, Hoekstra MR. Foodborne Illness Acquired in the United States—Unspecified Agents. Emerging Infectious Diseases. 2011; 17(1):16–22. https://doi.org/10.3201/eid1701.091101p2 PMID: 21192849

40. Hoffmann S, Batz MB, Morris JG Jr. Annual cost of illness and quality-adjusted life year losses in the United States due to 14 foodborne pathogens. J Food Prot. 2012; 75(7):1292–302. https://doi.org/10.4315/0362-028X.JFP-11-417 PMID: 22980013.

41. Scharff RL. Economic burden from health losses due to foodborne illness in the United States. J Food Prot. 2012; 75(1):123–31. Epub 2012/01/10. https://doi.org/10.4315/0362-028X.JFP-11-058 PMID: 22221364.

42. Wu HJ, Wu E. The role of gut microbiota in immune homeostasis and autoimmunity. Gut Microbes. 2012; 3(1):4–14. Epub 2012/02/24. https://doi.org/10.4161/gmic.19320 PMID: 22356853; PubMed Central PMCID: PMC3337124.

43. McNeilly TN, Mitchell MC, Rosser T, McAteer S, Low JC, Smith DG, et al. Immunization of cattle with a combination of purified intimin-531, EspA and Tir significantly reduces shedding of *Escherichia coli* O157:H7 following oral challenge. Vaccine. 2010; 28(5):1422–8. Epub 2009/11/12. https://doi.org/10.1016/j.vaccine.2009.10.076 PMID: 19903545.

44. Yoshida M, Claypool SM, Wagner JS, Mizoguchi E, Mizoguchi A, Roopenian DC, et al. Human neonatal Fc receptor mediates transport of IgG into luminal secretions for delivery of antigens to mucosal dendritic cells. Immunity. 2004; 20(6):769–83. Epub 2004/06/11. https://doi.org/10.1016/j.immuni.2004.05.007 PMID: 15189741.
